# Olanzapine Effects on Parvalbumin/GAD67 Cell Numbers in Layers/Subregions of Dorsal Hippocampus of Chronically Socially Isolated Rats

**DOI:** 10.3390/ijms242417181

**Published:** 2023-12-06

**Authors:** Andrijana Stanisavljević Ilić, Snežana Đorđević, Dragoš Inta, Stefan Borgwardt, Dragana Filipović

**Affiliations:** 1 Department of Molecular Biology and Endocrinology, “VINČA” Institute of Nuclear Sciences, National Institute of the Republic of Serbia, University of Belgrade, 11000 Belgrade, Serbia; andristanisavljevic@vinca.rs; 2 Poisoning Control Centre, Military Medical Academy, 11000 Belgrade, Serbia; ivezicnela@gmail.com; 3 Department for Community Health Faculty of Natural Sciences, Medicine, University of Fribourg, 1700 Fribourg, Switzerland; dragos.inta@unifr.ch; 4 Department of Biomedicine, University of Basel, 4001 Basel, Switzerland; 5 Department of Psychiatry and Psychotherapy, Center of Brain Behavior and Metabolism, University of Lübeck, 23562 Lübeck, Germany; stefan.borgwardt@uksh.de

**Keywords:** chronic social isolation, olanzapine, hippocampus, parvalbumin, GAD67

## Abstract

Depression is linked to changes in GABAergic inhibitory neurons, especially parvalbumin (PV) interneurons, which are susceptible to redox dysregulation. Olanzapine (Olz) is an atypical antipsychotic whose mode of action remains unclear. We determined the effect of Olz on PV-positive (+) and glutamate decarboxylase 67 (GAD67) + cell numbers in the layers of dorsal hippocampus (dHIPP) cornu ammonis (CA1–CA3) and dentate gyrus (DG) subregions in rats exposed to chronic social isolation (CSIS), which is an animal model of depression. Antioxidative enzymes and proinflammatory cytokine levels were also examined. CSIS decreased the PV+ cell numbers in the Stratum Oriens (SO) and Stratum Pyramidale (SP) of dCA1 and dDG. It increased interleukin-6 (IL-6), suppressor of cytokine signaling 3 (SOCS3), and copper–zinc superoxide dismutase (CuZnSOD) levels, and it decreased catalase (CAT) protein levels. Olz in CSIS increased the number of GAD67+ cells in the SO and SP layers of dCA1 with no effect on PV+ cells. It reduced the PV+ and GAD67+ cell numbers in the Stratum Radiatum of dCA3 in CSIS. Olz antagonizes the CSIS-induced increase in CuZnSOD, CAT and SOCS3 protein levels with no effect on IL-6. Data suggest that the protective Olz effects in CSIS may be mediated by altering the number of PV+ and GAD67+ cells in dHIPP subregional layers.

## 1. Introduction

It has been demonstrated that the dysfunction or loss of γ-aminobutyric acid (GABA) neurotransmission in brain regions has been implicated in psychiatric disease [1,2]. GABA is an inhibitory neurotransmitter [3] that provides an inhibitory regulation of brain neuronal activity. It is synthesized by glutamic acid decarboxylases (GAD65 and GAD67) [4] through glutamate decarboxylation, whereby GAD67 is constitutively active and produces over 90% of the basal level of GABA in the brain [4]. One of the brain’s most prevalent forms of GABA interneurons is parvalbumin (PV) interneurons, which are highly represented in the hippocampus (HIPP) [5]. These fast-spiking interneurons are characterized by their inhibitory feedback and feedforward perisomatic projections onto pyramidal neurons [6,7]. They have a specific distribution in the hippocampal CA1 and CA3 subregions within the Stratum Oriens (SO), the Stratum Pyramidale (SP), the Stratum Radiatum (SR), and none in the Stratum Lacunosum Moleculare (SLM) [8,9]. Also, they are localized in the molecular layer (ML) and granule cell layer (GCL) as well as in the hilus (H) of the DG. PV interneurons are highly vulnerable to chronic stress, whereby a reduced number of PV cells was found in the rat HIPP [10]. Our recent studies have shown that adult male rats following chronic social isolation (CSIS) which exhibited depressive- and anxiety-like behaviors also showed a reduced number of PV positive (+) cells in the subregions of the HIPP [11,12,13]. Importantly, this effect was reversed by chronic treatment with fluoxetine or tianeptine [14,15]. These findings suggest that reduced PV+ cell numbers and behavioral impairments associated with CSIS are casually linked. Postmortem studies have also reported that people with major depressive disorder (MDD) and bipolar disorder have a decreased number of PV interneurons in the HIPP and prefrontal cortex [16].

Functional consequences of chronic stress on PV cells have been observed in brain regions, particularly in the HIPP [11], as one of the brain regions involved in mood and anxiety disorders [17]. Its anatomical division of the subregions into distinct layers influences brain functions. Investigation of the changes in protein levels in the hippocampal subregions in a layer-specific manner may offer important details regarding the neuroanatomical role in development of disease and therapy outcomes. The dorsal (d)HIPP represents a center of spatial learning- and social-related memory processes [18]. A recent study has shown that alterations in the perinuclear network that surrounds PV+ interneurons in the dHIPP of adolescent mice following social isolation stress make them more vulnerable to stress [19]. Moreover, if energy demand and/or redox balance are dysregulated, these interneurons may have a major impact on cognitive decline [17]. Furthermore, dysregulated redox mechanisms may increase the release of proinflammatory cytokines such as interleukin (IL)-6, which was found in the blood samples of depressed patients [20]. IL-6 initiates a signaling pathway consisting of JAK/STAT proteins and characterized by a specific negative feedback loop by cytoplasmic protein, suppressor of cytokine signaling-3 (SOCS3) [21]. We have already demonstrated that the CSIS paradigm induced a depressive-like phenotype in adult male Wistar rats and upregulated the protein level of IL-6 in the hippocampus, while antidepressant fluoxetine treatment normalized the depressive phenotype and the CSIS-induced protein level of IL-6 [22].

Olanzapine (Olz) is a thienobenzodiazepine derivative classified as an atypical antipsychotic used to treat psychiatric disorders [23,24,25]. In addition to its antipsychotic efficacy, it also possesses antidepressant- and anxiolytic-like effects. Its antagonistic effect on the dopamine (D_2_) and 5-HT2_A_ receptors is thought to be the source of its antipsychotic properties [26]. Moreover, as an antagonist of 5-HT_2_ receptor, Olz increases the release of serotonin from the presynaptic membrane, resulting in an increase in serotonin activity at the 5HT_1_ receptor, which may account for its antidepressant and anxiolytic properties. [24]. In depressed patients, atypical antipsychotics are used as augmentation agents to improve the efficacy of antidepressants for treatment-resistant depression [27,28]. According to published data, Olz’s effect on the GABAergic system may be a factor in its ability to have anxiolytic-like effects [29]. Moreover, benzodiazepines enhance the inhibitory effect of GABA by the positive allosteric modulation of GABAa receptors [30], making them therapeutic candidates for mood disorders [31]. However, its mode of action is still unclear.

In the present study, we used CSIS (6 weeks) to investigate its detrimental effects on the PV+ and GAD67+ cell numbers in the layers within subregions of the rat dHIPP such as the SO, SP, SR and Stratum Lacunosum Moleculare (SLM) of dCA1-CA3, Molecular Layer-Granular Cell Layer (ML-GCL), and H of the dDG. The CSIS paradigm was chosen since it induces neurochemical, neuroendocrine and behavioral changes in rats similar to those observed in humans with psychiatric disorders, including depression [32] and schizophrenia [33]. Importantly, symptoms are reversible with antidepressant treatments [34,35,36,37]. We also investigated the effect of chronic Olz treatment (lasting 3 weeks of 6-week CSIS) in counteracting the CSIS-induced alterations in the number of PV+ and GAD67+ cells and its antioxidative and anti-inflammatory potential on the CSIS-induced changes. We hypothesized that the detrimental effect of CSIS or the antidepressive effect of Olz treatment could be related to changes in the number of PV+ and GAD67+ cells in the layers within subregions of the rat dHIPP, which is associated with antioxidative defense and the release of proinflammatory cytokines. Hence, we determined the protein levels of antioxidative enzymes copper–zinc superoxide dismutase (CuZnSOD) and catalase (CAT). Since the exposure of rats to CSIS has been shown to induce inflammation in the HIPP [22], the protein levels of IL-6 and SOCS3 were also determined.

## 2. Results

### 2.1. Behavior Testing—Forced Swim Test

Statistically significant results of immobility, swimming, and climbing time in the forced swim test (FST) at weeks 0, 3, and 6 are presented in Table 1.

Regarding immobility time, a three-way repeated measures ANOVA revealed the significant main effects of CSIS (F_1.20_ = 8.99, *p* < 0.01) and time (F_2.40_ = 10.12, *p* < 0.001) as well as the effects of the time × CSIS interaction (F_2.40_ = 3.86, *p* < 0.05) and time × Olz interaction (F_2.40_ = 3.65, *p* < 0.05). A three-way repeated measures ANOVA for swimming time revealed significant main effects of CSIS (F_1.20_ = 15.89, *p* < 0.001) and time (F_2.40_ = 6.81, *p* < 0.01) as well as the effects of the time × CSIS interaction (F_2.40_ = 3.32, *p* < 0.05) and time × Olz interaction (F_2.40_ = 10.38, *p* < 0.001). Regarding climbing time, a three-way repeated measures ANOVA revealed a significant main effect of time (F_2.40_ = 50.7, *p* < 0.001) and the effect of the time × Olz interaction (F_2.40_ = 4.54, *p* < 0.05).

For immobility time, a significant increase in CSIS at 3-week and 6-week tests compared to CSIS (baseline) (** *p* < 0.01; *** *p* < 0.001, respectively) was revealed. A significant decrease was seen in CSIS + Olz (6 weeks) vs. CSIS (6 weeks), ^##^ *p* ≤ 0.01. For swimming time, a significant increase in CSIS + Olz (6 weeks) vs. CSIS (6 weeks), ^##^ *p* < 0.01 was found. A significant decrease was seen in CSIS (3 weeks) vs. CSIS (baseline), * *p* < 0.05 and CSIS (6 weeks) vs. CSIS (baseline), * *p* < 0.05. For climbing time, a significant decrease in Cont (6 weeks) vs. Cont (baseline), ** *p* < 0.01, CSIS (3 weeks) vs. CSIS (baseline), ** *p* < 0.01, and CSIS (6 weeks) vs. CSIS (baseline), *** *p* < 0.001 were revealed.

### 2.2. Layer-Specific Distribution of the Number of PV+ and GAD67+ Cells in the dCA1 Subregion

**Figure 1:**
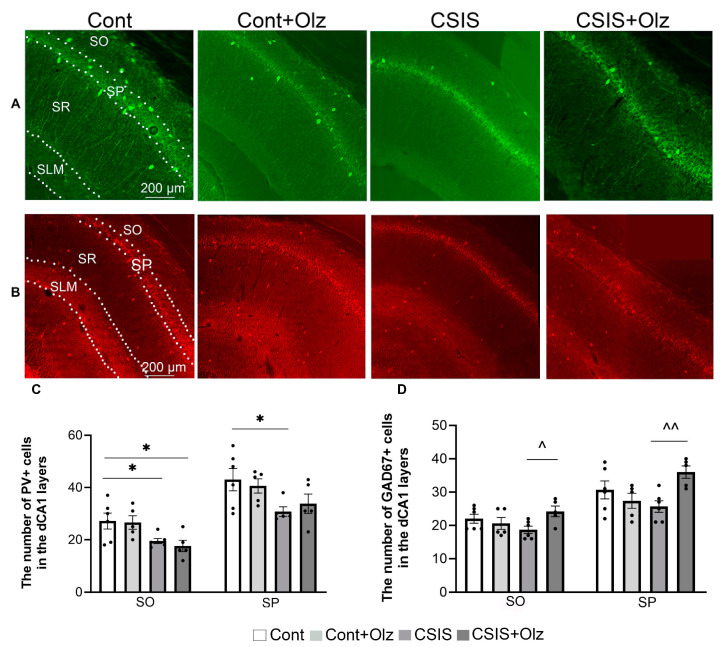
Representative photomicrographs of fluorescent PV+ cells (green) (**A**) and GAD67+ cells (red) (**B**) in the Stratum Oriens (SO), Stratum Pyramidale (SP), Stratum Radiatum (SR), and Stratum Lacunosum Moleculare (SLM) layers of the dCA1 subregion. A statistically significant change in the number of PV+ cells (**C**) and GAD67+ cells (**D**) in the SO and SP layers of the dCA1 subregion in controls (Cont), olanzapine-treated controls (Cont + Olz), chronic social isolation (CSIS), and olanzapine-treated CSIS (CSIS + Olz). Significant differences between groups obtained by a two-way ANOVA followed by Duncan’s post hoc test are indicated as follows: * *p* < 0.05 comparisons always against controls; ^ *p* < 0.05, ^^ *p* < 0.001 comparisons between CSIS + Olz and CSIS groups. The results are presented as mean ± standard error of mean (SEM) of 5–6 rats per group. Scale bar 200 µm.

A statistically significant change in the number of PV+ and GAD67+ cells in the SO and SP of the dCA1 is presented in Figure 1.

A two-way ANOVA revealed a significant main effect of CSIS (F_1.18_ = 12.61, *p* < 0.01; F_1.17_ = 7.63, *p* ≤ 0.01) for the number of PV+ cells in the SO and SP layers, respectively. A significant decrease in the number of PV+ cells in CSIS or CSIS +Olz as compared to controls (* *p* < 0.05) in the SO layer was found. In the SP layer, a significant decrease in these cells was seen in CSIS as compared to controls (* *p* < 0.05) (Figure 1C). No significant changes in the number of PV+ cells in the SR layer were observed, while no PV+ cells were detected in the SLM.

A two-way ANOVA showed a significant effect of Olz × CSIS interaction (F_1.18_ = 5.8, *p* < 0.05; F_1.18_ = 8.97, *p* < 0.01) in the SO and SP layers, respectively, for the number of GAD67+ cells. A significant increase in the number of these cells in Olz-treated CSIS as compared to CSIS (^ *p* < 0.05, ^^ *p* < 0.01) rats, in the SO and SP layers, respectively, was found (Figure 1D). There was no significant effect on the number of GAD67+ cells between experimental groups in the SR and SLM layers.

### 2.3. Layer-Specific Distribution of the Number of PV+ and GAD67+ Cells in the CA2 Subregion

**Figure 2:**
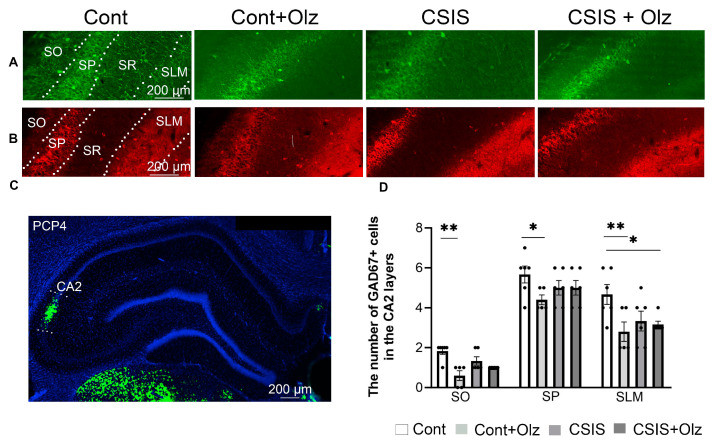
Representative photomicrographs of fluorescent PV+ cells (green) (**A**) and GAD67+ cells (red) (**B**) in the Stratum Oriens (SO), Stratum Pyramidale (SP), Stratum Radiatum (SR), and Stratum Lacunosum Moleculare (SLM) layers of CA2 subregion. (**C**) Purkinje cell protein 4 (PCP4) immunostaining in the CA2 subregion (green) in photomicrograph of DAPI-stained nuclei (blue) in the coronal rat brain section of dHIPP. A statistically significant change in the number of GAD67+ cells in SO, SP and SLM layers (**D**) of the CA2 subregion in controls (Cont), olanzapine-treated controls (Cont + Olz), chronic social isolation (CSIS), and olanzapine-treated CSIS (CSIS + Olz). Significant differences between groups obtained by a two-way ANOVA followed by Duncan’s post hoc test are indicated as follows: * *p* < 0.05, ** *p* ≤ 0.01 comparisons always against controls. The results are presented as mean ± standard error of mean (SEM) of 5–6 rats per group. Scale bar 200 µm.

A statistically significant change in the number of GAD67+ cells in the SO, SP, and SLM layers of the CA2 subregion is presented in Figure 2.

A two-way ANOVA revealed no significant difference in the number of PV+ cells between the experimental groups in the SO and SP layers. Moreover, in the SR and SLM layers, PV+ cells were not detected (Figure 2A).

A two-way ANOVA revealed a significant main effect of Olz (F_1.19_ = 9.63, *p* < 0.01; F_1.19_ = 6.41, *p* < 0.05) in the SO and SLM, respectively, for the number of GAD67+ cells. A significant decrease in the number of GAD67+ cells was revealed in Olz-treated controls as compared to controls (** *p* ≤ 0.01, * *p* < 0.05, ** *p* ≤ 0.01) in the SO, SP and SLM layers, respectively (Figure 2D), as well as in Olz-treated CSIS rats as compared to controls (* *p* < 0.05) in the SLM layer (Figure 2D). No significant effect was seen on the number of GAD67+ cells between experimental groups in the SR layer.

### 2.4. Layer-Specific Distribution of the Number of PV+ and GAD67+ Cells in the dCA3 Subregion

**Figure 3:**
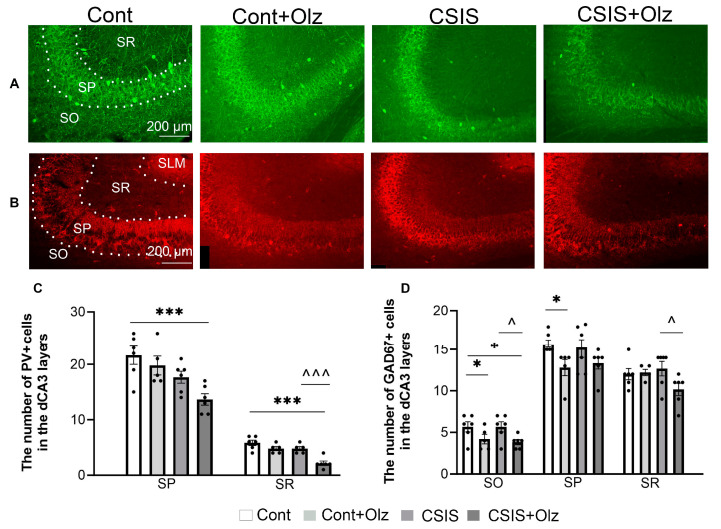
Representative photomicrographs of fluorescent PV+ cells (green) (**A**) and GAD67+ cells (red) (**B**) in the Stratum Oriens (SO), Stratum Pyramidale (SP), Stratum Radiatum (SR), and Stratum Lacunosum Moleculare (SLM) layers of the CA3 subregion. A statistically significant change in the number of PV+ cells in the SP and SR layers (**C**), and GAD67+ cells in the SO, SP and SR layers (**D**) of the dCA3 subregion in controls (Cont), olanzapine-treated controls (Cont + Olz), chronic social isolation (CSIS), and olanzapine-treated CSIS (CSIS + Olz). Significant differences between groups obtained by a two-way ANOVA followed by Duncan’s post hoc test are indicated as follows: * *p* ≤ 0.05, *** *p* ≤ 0.001, comparisons always against controls; ^ *p* < 0.05, ^^^ *p* < 0.001, comparisons between CSIS + Olz and CSIS The results are presented as mean ± standard error of mean (SEM) of 5–6 rats per group. Scale bar 200 µm.

A statistically significant change in the number of PV+ and GAD67+ cells in the SO, SP, and SR layers of the dCA3 is presented in Figure 3.

A two-way ANOVA revealed a significant main effect of CSIS (F_1.19_ = 13.59, *p* < 0.01; F_1.18_ = 15.99, *p* < 0.001) in the SP and SR layers, respectively, as well as a significant main effect of Olz (F_1.18_ = 18.53, *p* < 0.001) in the SR layer for the number of PV+ cells. A significant decrease was seen in Olz-treated CSIS rats as compared to controls (*** *p* ≤ 0.001) in the SP and SR layers (Figure 3C) as well as between Olz-treated CSIS vs. CSIS rats (^^^ *p* < 0.001) in the SR layer (Figure 3C). No significant change in the number of PV+ cells between the experimental groups in the SO layer was observed. We did not detect PV+ cells in the SLM layer.

A two-way ANOVA revealed a significant main effect of Olz (F_1.19_ = 11.80, *p* < 0.01; F_1.19_ = 6.84, *p* < 0.05) in the SO and SP layers, respectively, on the number of GAD67+ cells. A significant decrease in the number of these cells in Olz-treated controls as compared to controls (* *p* < 0.05, * *p* ≤ 0.05) in the SO and SP layers, respectively (Figure 3D), was detected. A significant decrease was seen in Olz-treated CSIS rats as compared to controls (* *p* < 0.05) and CSIS (^ *p* < 0.05) in the SO layer (Figure 3D). Moreover, there was a significant decrease in Olz-treated CSIS rats as compared to CSIS (^ *p* < 0.05) in the SR layer (Figure 3D). No significant change in the number of GAD67+ cells between experimental groups in the SLM layer was found.

### 2.5. The Number of PV+ and GAD67+ Cells in the ML-GCL and H of the dDG Subregion

**Figure 4:**
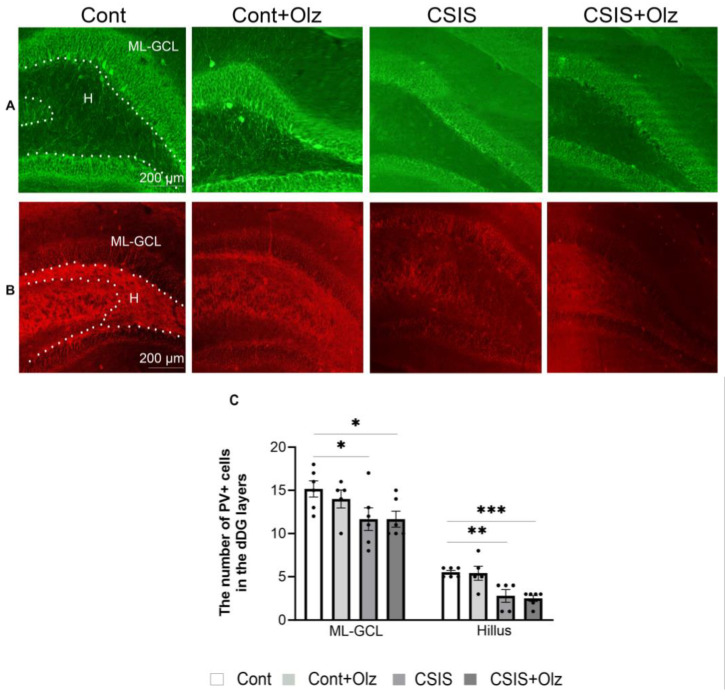
Representative photomicrographs of fluorescent PV+ cells (green) (**A**) and GAD67+ cells (red) (**B**) in the Molecular Layer-Granular Cell Layer (ML-GCL) and Hillus (H) of the dDG. A statistically significant change in the number of PV+ cells in the ML-GCL and Hillus (H) (**C**) of the dDG subregion in controls (Cont), olanzapine-treated controls (Cont + Olz), chronic social isolation (CSIS), and olanzapine-treated CSIS (CSIS + Olz). Significant differences between groups obtained by a two-way ANOVA followed by Duncan’s post hoc test are indicated as follows: * *p* < 0.05, ** *p* < 0.01, *** *p* < 0.001 comparisons always against controls. The results are presented as mean ± standard error of mean (SEM) of 5–6 rats per group. Scale bar 200 µm.

A statistically significant change in the number of PV+ cells in the ML-GCL and H of the dDG is presented in Figure 4.

A two-way ANOVA revealed a significant main effect of CSIS (F_1.19_ = 8.81, *p* < 0.01; F_1.18_ = 27.42, *p* < 0.001) in ML-GCL and H, respectively, for the number of PV+ cells. A significant decrease was seen in the CSIS and Olz-treated CSIS rats as compared to controls in the ML-GCL (* *p* < 0.05) and in H (** *p* < 0.01, *** *p* < 0.001), respectively (Figure 4C). A two-way ANOVA revealed no significant effect in the number of GAD67+ cells in the ML-GCL and H.

### 2.6. The Number of PV+ and GAD67+ Cells in dCA1, CA2, dCA3, and dDG Subregions

**Figure 5:**
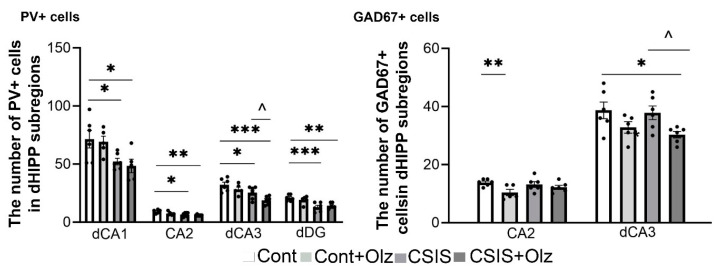
A statistically significant change in the number of PV+ cells in the dCA1, CA2, dCA3, and dDG subregions as well as in the GAD67+ cells in CA2 and dCA3, in controls (Cont), olanzapine-treated controls (Cont + Olz), chronic social isolation (CSIS), and olanzapine-treated CSIS (CSIS + Olz). Significant differences between groups obtained by a two-way ANOVA followed by Duncan’s post hoc test are indicated as follows: * *p* < 0.05, ** *p* < 0.01, *** *p* < 0.001 comparisons always against controls; ^ *p* < 0.05, CSIS + Olz vs. CSIS. The results are presented as mean ± standard error of mean (SEM) of 5–6 rats per group.

A statistically significant change in the numbers of PV+ and GAD67+ cells in the dHIPP subregions (dCA1, CA2, dCA3, and dDG) is presented in Figure 5 (left and right, respectively).

A two-way ANOVA showed significant main effects of CSIS (F_1.18_ = 12.36, *p* < 0.01; F_1.18_ = 7.46, *p* ≤ 0.01; F_1.19_ = 14.69, *p* ≤ 0.001; F_1.19_ = 24.04, *p* < 0.001) in the dCA1, CA2, dCA3, and dDG, respectively, and Olz (F_1.19_ = 6.34, *p* < 0.05) in the dCA3 for the number of PV+ cells. A significant decrease was seen in the CSIS and Olz-treated CSIS rats as compared to controls in the dCA1 (* *p* < 0.05), CA2 (* *p* < 0.05, ** *p* < 0.01), dCA3 (* *p* < 0.05, *** *p* < 0.001), and dDG subregions (*** *p* < 0.001, ** *p* < 0.01), respectively (Figure 5, PV+ cells). In addition, a significant decrease was seen in Olz-treated CSIS as compared to CSIS rats in the dCA3 subregion (^ *p* < 0.05) (Figure 5, PV+ cells).

Regarding the changes in the number of GAD67+ cells in the dCA1, CA2, dCA3 and dDG subregions, a two-way ANOVA revealed the effects of the Olz × CSIS interaction (F_1.19_ = 4.85, *p* < 0.05) in the dCA1 and Olz treatment (F_1.19_ = 8.83, *p* < 0.01, F_1.19_ = 9.63, *p* < 0.01) in CA2 and dCA3, respectively. A significant decrease was found in the Olz-treated rats as compared to controls (** *p* < 0.01) in the CA2 (Figure 5, GAD67+ cells) as well as in the Olz-treated CSIS rats as compared to controls (* *p* < 0.05) and CSIS rats (^ *p* < 0.05) in the dCA3 (Figure 5, GAD67+ cells). No significant effect was seen in the dCA1 and dDG.

### 2.7. Western Blot Analyses

**Figure 6:**
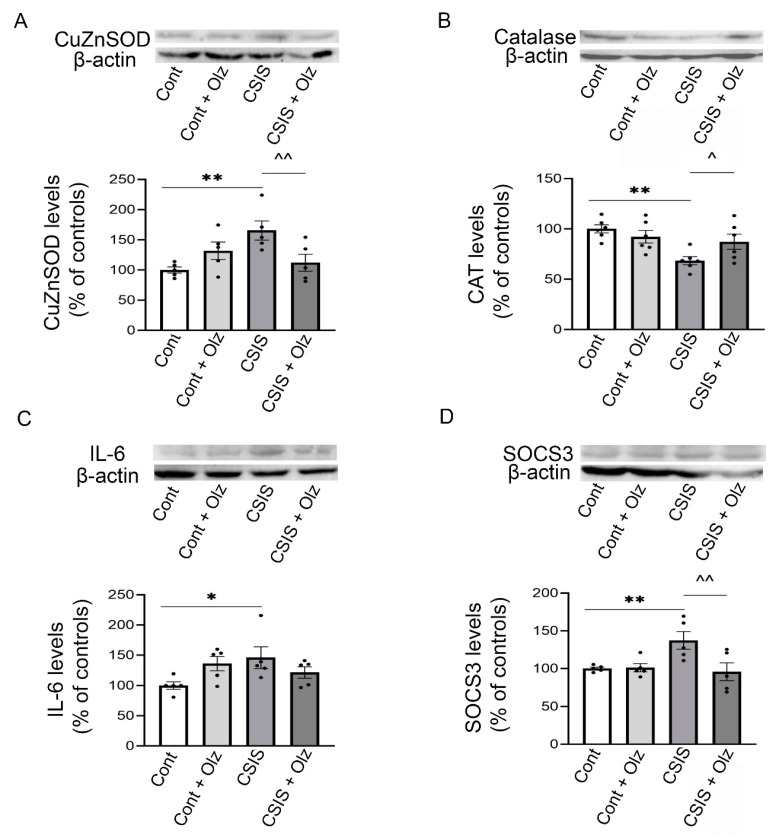
The cytosolic protein levels of CuZnSOD (**A**), CAT (**B**), IL-6 (**C**) and SOCS3 (**D**) in the HIPP of controls (Cont), olanzapine-treated controls (Cont + Olz), chronic social isolation (CSIS), and olanzapine-treated CSIS (CSIS + Olz). Significant differences between groups obtained by a two-way ANOVA followed by Duncan’s post hoc test are indicated as follows: (**A**) CuZnSOD protein levels—** *p* < 0.01, CSIS vs. Cont; ^^ *p* ≤ 0.01, CSIS + Olz vs. CSIS. (**B**) CAT protein levels—** *p* < 0.01, CSIS vs. Cont; ^ *p* < 0.05, CSIS + Olz vs. CSIS. (**C**) IL-6 protein levels—* *p* < 0.05, CSIS vs. Cont. (**D**) SOCS3 protein levels—** *p* ≤ 0.01, CSIS vs. Cont; ^^ *p* < 0.01, CSIS + Olz vs. CSIS). The results are presented as the mean ± standard error of mean (SEM) of 5–6 independent measurement per group.

We examined the cytosolic protein levels of CuZnSOD, CAT, IL-6, and SOCS3 of the rat HIPP (Figure 6A–D, respectively). A two-way ANOVA revealed a significant main effect of Olz × CSIS interaction (F_1.16_ = 10.54, *p* < 0.01) on cytosolic CuZnSOD protein levels. A post hoc test revealed a significant increase in CuZnSOD protein levels in CSIS rats as compared to controls (** *p* < 0.01) as well as a significant decrease in Olz-treated CSIS rats as compared to CSIS rats (^^ *p* ≤ 0.01) (Figure 6A). For the cytosolic CAT protein levels, a two-way ANOVA revealed a significant main effect of CSIS (F_1.20_ = 10.32, *p* < 0.01) and effect of Olz × CSIS interaction (F_1.20_ = 5.5, *p* < 0.05). A post hoc test revealed a significant decrease in CAT protein levels in the CSIS rats as compared to controls (** *p* < 0.01) as well as a significant increase in Olz-treated CSIS rats as compared to CSIS rats (^ *p* < 0.05) (Figure 6B). A two-way ANOVA revealed a significant main effect of Olz treatment × CSIS interaction (F_1.16_ = 6.17, *p* < 0.05) on the cytosolic IL-6 protein levels. A post hoc test revealed a significant increase in the IL-6 protein levels of CSIS rats as compared to controls (* *p* < 0.05) (Figure 6C). For the SOCS3 protein levels, a two-way ANOVA revealed a significant main effect of Olz (F_1.16_ = 5.35, *p* < 0.05) and effect of Olz × CSIS interaction (F_1.16_ = 5.91, *p* < 0.05). A post hoc test revealed a significant increase in SOCS3 protein levels in CSIS rats as compared to controls (** *p* ≤ 0.01), and a significant decrease in Olz-treated CSIS rats as compared to CSIS rats (^^ *p* < 0.01) (Figure 6D). The results are presented as the mean ± standard error of mean (SEM) of 5–6 independent measurements per group. The blots that are shown are representative, cropped images of several Western blots that have been carried out. Full-length Western blot images are included in the Supplementary Materials files.

## 3. Discussion

Chronic psychosocial stress in adulthood modulates brain structure and function, thus increasing the risk for psychiatric disorders [38]. In our study, we used the CSIS paradigm and/or chronic Olz treatment to investigate their effects on the PV+ and GAD67 + cell numbers in the layers of dHIPP and the potential contribution of antioxidative enzymes as well as the proinflammatory cytokine IL-6, which has been associated with the pathophysiology of psychiatric disorders [39]. Results demonstrated that rats following CSIS show depressive-like behavior as assessed by immobility time in the FST. The findings are consistent with prior research showing that socially isolated rats exhibit depression-like behavior [40,41]. Additionally, CSIS also induced anxiety-like behavior in these rats, which was assessed by marble burring [42], open field [41] and elevated plus maze [43] tests. Chronic treatment with Olz reversed depressive-like behavior in CSIS rats, indicating its antidepressant-like activity.

CSIS induced a decrease in the number of PV+ cells in dHIPP subregions, confirming earlier data [14,44] without changes in the number of GAD67+ cells. A decreased number of PV+ cells in the SO (72%) and SP (72%) layers (no effect on SR layer) of dCA1 and both dDG layers (ML-GCL-76% and H-54%) compared to the control may result in decreased GABAergic inhibitory control. The obtained results indicate dDG as a possible target subregion for a 6-week CSIS. The reduced number of PV+ cells might cause excessive glutamate release, leading to neuronal hyperactivity and neurotoxicity [45] as well as a reduction in inhibitory synaptic transmission that affects cognition [46]. Thus, rats exposed to the chronic mild stress paradigm showed decreased PV expression, which contributed to impaired cognitive function [47]. The report by Czéh et al. [11] using the paradigm of nine weeks of mild chronic stress also suggests a decreased number of PV interneurons around 20–35% in CA subregions of the dHIPP. A reduction in the number of these interneurons in our study may be related to the stress-induced suppression of adult neurogenesis [48,49], since about 14% of newly generated neurons in rat DG are GABAergic PV+ cells [50], which participate in the regulation of adult neurogenesis [51]. Moreover, the hippocampal DG has been shown to play a role in the pathogenesis of MDD [52]. In addition, compromised maturation or a reduced expression of PV (or to undetectable levels), as measured by immunofluorescence in the absence of neuronal loss, may be the reasons for the reduced number of PV+ cells. This result is supported by data showing that CSIS does not increase caspase-3 protein expression in dHIPP rats [53].

PV interneurons exhibit fast spiking activity, which imposes a high amount of energy and a higher number of mitochondria and cytochrome c [54], exposing them to the potentially toxic effects of reactive oxidative stress and causing a redox dysregulation of the cells [17]. Previous study has shown redox dysregulation in the HIPP of adult male socially isolated rats [22], which may structurally damage the PV interneurons and cause a reduced number of PV+ cells. Therefore, we decided to investigate the protein levels of the antioxidative enzymes CuZnSOD and CAT. We found that the CSIS-induced increase in cytosolic CuZnSOD and decrease in CAT protein levels can be attributed to oxidative stress, which requires higher CuZnSOD activity in order to protect cells against oxidative stress. Additionally, higher SOD activity could result in increased hydrogen peroxide production, providing more substrate for CAT. However, the observed decrease in CAT protein levels may be due to the inability to remove hydrogen peroxide and its accumulation, leading to an increase in hippocampal oxidative stress. Oxidative stress is tightly connected to inflammation and the expression of interleukins (IL-6, IL-1β) [55]. It was noted that IL-6 mediated the loss of PV-expressing GABAergic interneurons [56]. In agreement with that, we found a CSIS-induced increase in IL-6 and SOCS3 protein levels in the HIPP without any suppressive effect of the feedback-loop inhibition. Similar results were obtained with the research of Rossetti and colleagues [57], who explained that dysregulation of the SOCS3-dependent inhibition loop pathway in mild chronic stress might be due to the long-lasting overactivation of IL-6 signaling, which leads to the creation of a chronically damaging environment, and the lack of physiological blocking and adaptive reactions. Moreover, an increase in brain production of IL-6 under pathological conditions can decrease adult neurogenesis in the DG of HIPP [55], which can mediate a decrease in the number of PV+ cells in the dDG, as we found in our study.

Conversely, Olz treatment in CSIS rats exerted effects on GAD67+ cells, which was revealed in the dCA1 (SO/SP) and dCA3 (SO/SR) as compared to CSIS rats. A decreased number of PV+ and GAD6+ cells in dCA3 within the SR layer was found. Contrary to that, the increased number of GAD67+ cells in the SO and SP layers of dCA1 in Olz-treated CSIS rats compared to CSIS may represent Olz’s effectiveness by enhancing inhibitory synaptic neurotransmission. This finding is supported by data showing that chronic treatment with the antipsychotic lurasidone normalizes the chronic mild stress-induced decrease in PV expression in dHIPP [58]. The antipsychotic drug clozapine also regulates the function of GABAergic interneurons in dHIPP by preventing the CSIS-induced decrease in the number of PV+ cells in CA2/3 and DG subregions [44]. In addition to PV+ cells, an increase in GAD67+ cell numbers was also found in the dHIPP (dCA1/dCA3) of CSIS rats treated with the antidepressant tianeptine [14]. Although Olz failed to restore the CSIS-reduced PV+ cell numbers in the dCA1, CA2 and dDG, it prevented its further decrease with the exception of the previously mentioned dCA3 and its SR layer as compared to CSIS. Additionally, Olz showed no significant effect on the number of PV+ cells within dHIPP subregions in controls. We also found that Olz antagonized the CSIS-induced increase in hippocampal CuZnSOD levels and decrease in CAT levels, suggesting its ability to modulate oxidative stress, which may contribute to its therapeutic effects. SOCS3 protein levels were decreased by Olz treatment with no effect on IL-6. Moreover, an insufficient reduction in IL-6 protein levels could lead to Olz’s inability to restore reduced PV+ cell numbers in the CSIS rats. However, there is a possibility that the Olz treatment achieved its protective effects through other calcium-binding proteins such as calretinin or calbindin. Hence, additional research is required to fully comprehend the role of IL-6 signaling and other GABAergic interneurons.

In conclusion, chronically socially isolated rats showed a decreased number of PV+ cells in distinct layers of CA1 and DG subregions of dHIPP along with an altered antioxidative defense system and increased proinflammatory cytokine IL-6 and SOCS3, which could be, at least in part, a potential mechanism mediating the dysfunction of these cells. Chronic Olz treatment normalized a decrease in the number of PV+ cells in the layers of SO and SP of dCA1 and ML-GCL and H of dDG in dHIPP, with the exception of the SR layer in dCA3, and it modulated the number of GAD67+ cells in the SO/SP layers of dCA1 and the SO/SR layers of dCA3. Also, Olz reversed the CSIS-induced changes in the protein levels of CuZnSOD, CAT, and SOCS3 but did not reverse the increased IL-6 protein levels. Data indicate that effective Olz treatments in CSIS could be associated with a modulated number of PV + and GAD67 + cells in certain layers of dHIPP subregions, whereby antioxidative defense might be beneficial to PV networks.

## 4. Material and Methods

### 4.1. Animals

Adult male Wistar rats (2–2.5 months old, body weight 300–350 g) were housed under standard conditions in groups of four per cage and maintained under standard conditions of a temperature of 20 ± 2 °C with access to water and food (commercial rat pellets) ad libitum. Rats were maintained under a 12 h light/dark cycle (lights on from 07.00 to 19.00 h). Experimental procedures were realized in accordance with the Ethical Committee for the Use of Laboratory Animals of the “VINČA” Institute of Nuclear Sciences, University of Belgrade, National Institute of the Republic of Serbia, which follows the guidelines of the registered “Serbian Society for the Use of Animals in Research and Education”, license 323-07-02256/2019-05.

### 4.2. Olanzapine Solution

Olz tablets (containing 10 mg of Olz) were crushed to a fine powder and dissolved in 0.1 M HCl; pH was adjusted to 5.8 with 1 M NaOH with the aid of ultrasound and filtered through Whatman No. 42 filter paper [59]. Rats received a daily intraperitoneal (i.p.) injection of 7.5 mg/kg of olanzapine–hydrochloride (hereafter referred to as Olz) or vehicle (0.1 M HCl, pH 5.8) during the last three weeks of a six-week experimental procedure. Olz concentration in the serum samples was determined by liquid chromatography-mass spectrometry. Serum concentrations of Olz are within the range 102 to 116 ng/mL (SEM ± 1.88) in the Olz-treated controls and 110 to 138 ng/mL (SEM ± 4.32) in the Olz-treated CSIS rats, which are in agreement with the data of Kapur and colleagues [60]. A chosen dose of Olz produces plasma levels in the rats that correspond to therapeutically effective concentrations in humans [61,62,63,64]. The rats were weighed once a week for calculations of the volume of Olz solution needed to be administered.

### 4.3. Study Design

**Figure 7:**
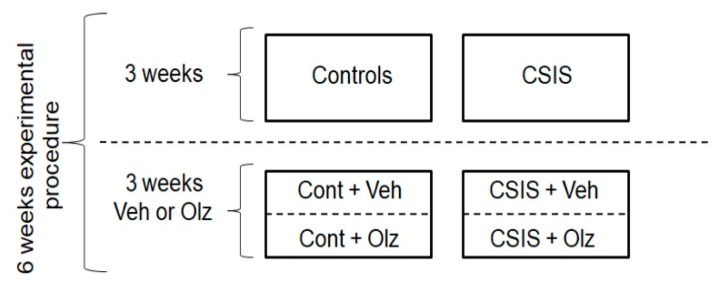
Schematic presentation of the study design. CSIS—chronic social isolation, Veh—vehicle, Olz—olanzapine.

At the beginning of the experiment, rats (*n* = 64) were divided randomly into control rats (*n* = 28), housed in groups up to three per cage, and individually housed rats (*n* = 36) in cages that underwent CSIS stress for six weeks. CSIS rats were deprived of any visual and tactile contacts but had regular auditory and olfactory experiences, according to the model of Garzón and Del Rio [65]. During the last three weeks of the experiment, both control and CSIS groups were further divided into matched subgroups and were administered daily with i.p. injections of vehicle (Cont + Veh, CSIS + Veh) or Olz (7.5 mg/kg/day) (Cont + Olz, CSIS + Olz). Control rats were housed in a separate room and had no contact with the CSIS rats. Behavior test results were used for rat segregation into the CSIS group (an increase in the immobility of >20%) and rats responding effectively to Olz treatment, i.e., the CSIS + Olz group (decline in immobility > 20%). Of these, 2 rats from the Control group, 3 rats from Control + Olz, 6 rats from the CSIS group (CSIS resilient), and 6 rats from the CSIS + Olz group (resilient to Olz treatment) were excluded, since they did not show relevant behavior. For the immunofluorescence analyses, we used 23 rats distributed into 4 groups (Cont, *n* = 6; Cont + Olz, *n* = 5; CSIS, *n* = 6; CSIS + Olz, *n* = 6). For the Western blot analyses, we used 24 rats distributed into 4 groups (Cont, *n* = 6; Cont + Olz, *n* = 6; CSIS, *n* = 6; CSIS + Olz, *n* = 6). The schematic presentation of the study design is given in Figure 7.

### 4.4. Behavioral Test

#### Forced Swim Test

FST was utilized to evaluate depressive-like behavior and antidepressant-like effects of Olz [66]. Rats were positioned inside a vertical cylindrical plexiglass container (height 45 cm × diameter 28 cm) that was 30 cm filled with tap water at 24 ± 1 °C. The test stage lasts for 5 min and is recorded by a Sony HDR-PJ410 camera. The behaviors scored in the FST were immobility, climbing, and swimming [67]. We performed FST at the beginning (baseline), in the middle (3 weeks), and at the end of the experiment (6 weeks). We used immobility time as a measure of behavioral despair [68]. Test results were analyzed by two observers blinded to experimental conditions.

### 4.5. Immunofluorescence

Twenty-four hours after the last Olz treatment, rats were deeply anesthetized with ketamine/xylazine 100/5 mg/kg (i.p.), transcardially perfused with physiological saline, and then sacrificed by guillotine decapitation (Harvard Apparatus, South Natick, MA, USA). The brains were removed and post-fixed overnight in 4% paraformaldehyde (pH 7.4) (Sigma Aldrich, St. Louis, MO, USA). Coronal sections of 40 µm thickness were cut using a vibrato (VT 100 S; Leica, Bensheim, Germany).

**Figure 8:**
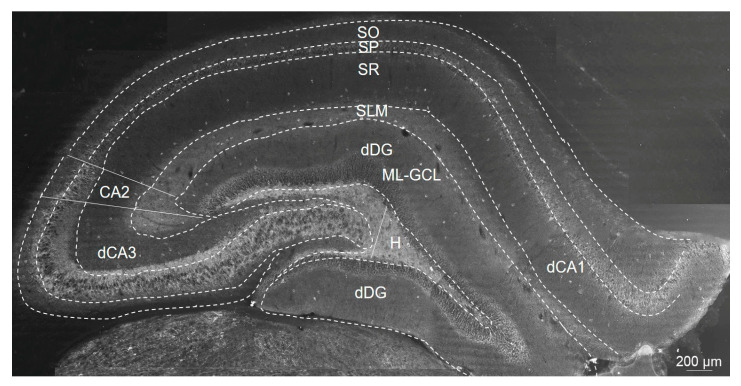
Representative photomicrograph of black–white parvalbumin positive (PV+) cells (scale bar 200 μm) with labeled dCA1, CA2, dCA3 and dDG regions, the Stratum Oriens (SO), Stratum Pyramidale (SP), Stratum Radiatum (SR), and Stratum Lacunosum Moleculare (SLM) layers of the dCA1-3 subregions, and the Molecular Layer-Granular Cell Layer (ML-GCL) and Hillus (H) of the dDG subregion in the coronal rat brain sections of dHIPP.

Free-floating coronal sections of the dHIPP (Bregma −3.12 to −3.60 mm) [69] were used for PV+ and GAD67+ cell immunofluorescence staining. Counting of the PV+ and GAD67+ cell numbers was performed in the SO, SP, SR, and SLM layers of dCA1-3 as well as in the ML-GCL and H layers of dDG and subregions of the rat dHIPP (presented in Figure 8). Identification of the CA2 region was completed by immunostaining with Purkinje cell protein 4 (PCP4), which delineates borders between CA3/CA2 and CA2/CA1 borders [44,70,71]. Brain sections were washed in 0.1 M PBS, pH 7.4, and incubated for approximately 1 h at room temperature (RT) in a blocking solution containing 5% normal donkey serum (NDS, Jackson ImmunoResearch, West Grove, PA, USA, 017-000-121) and 0.3% Triton X-100 in PBS. Brain sections were first incubated with a primary antibody, mouse anti-GAD67 (Millipore, Burlington, MA, USA, Cat# MAB5406B, RRID: AB_2938602, 1:100), and dissolved in a blocking solution. After being washed several times in PBS, sections were exposed to a rabbit anti-PV primary antibody (Swant, Burgdorf, Switzerland, Cat# PV27, RRID:AB_2631173, 1:500) for 24 h at 4 °C, which was followed by secondary antibody exposure (Alexa 555, donkey anti-mouse IgG, Thermo Fisher Scientific, Waltham, MA, USA, Cat #A-31570, RRID:AB_2536180, 1:500, and Alexa 488-conjugated donkey anti-rabbit IgG, Thermo Fisher Scientific Cat #A-21206, RRID:AB_2535792, 1:1000, for GAD67 and PV, respectively) for 2 h (each) at RT in a 0.1 M PBS solution containing 5% NDS. Sections were counterstained with 100 ng/mL DAPI (4′-6-diamidino-2-phenylindole, Vector Laboratories, Newark, CA, USA) and then mounted on slides and cover-slipped with fluorescent mounting medium (Dako North America, Inc. Carpinteria, CA, USA). Images of brain sections were visualized using a ZEISS microscope (Jena, Germany) equipped with the camera AxioCAM. dHIPP sections of 5–6 rats per group, in triplicate per rat, were analyzed. Averaged triplicate values per rat were taken as 5–6 independent values that were further statistically analyzed. We counted PV+ and GAD67+ cell numbers using ImageJ software (version 1.51 w). The counting was performed separately for each layer inside the borders of entire subregions.

### 4.6. Preparation of Cytosolic Fractions from the Rat Hippocampus

Rats were anesthetized with ketamine/xylazine 100/5 mg/kg (i.p.) 24 h following the final Olz treatment, perfused with physiological saline, and then sacrificed by guillotine decapitation (Harvard Apparatus, South Natick, MA, USA). The whole brain was immediately removed, and the HIPP was dissected on ice. Using a Potter–Elvehjem glass homogenizer and a Teflon pestle, hippocampi were homogenized at 4 °C by 12 strokes at 800 rpm in homogenization buffer (0.25 M sucrose, 10 mM Tris/HCl (pH 7.4)) that contained protease inhibitor cocktail tablets (complete tablets, Mini, EASY pack, Roche, Mannheim, Germany). Homogenates were centrifuged at 3300 rpm (Beckman, CA, USA, JA-20) for 10 min at 4 °C to obtain the crude nuclear pellet and the S1 supernatant. The remaining nuclei were then removed from the supernatant by repeating centrifugation under the same conditions. To obtain the mitochondrial pellet, the S1 supernatant was centrifuged at 18,000 rpm (Beckman, JA-20) for 20 min. To achieve pure cytosolic fractions, the resulting supernatant (S2) was subsequently centrifuged at 33,000 rpm (Beckman, Type Ti 50) for 40 min at 4 °C [22]. The Lowry method [72] was used to measure the protein concentrations, using purified bovine serum as a standard.

### 4.7. Western Blot Analysis

The cytosolic fractions of the hippocampi (40 µg) were separated on sodium dodecyl-sulfate–polyacrylamide gel electrophoresis and transferred to a polyvinylidene difluoride membrane (Immobilion Transfer Membrane) using a Mini Trans-blot device (Bio-Rad, Hercules, CA, USA) [22]. Membranes were further blocked in 5% bovine serum albumin (BSA, ROTH) in Tris-buffered saline (TBS) (SERVA) (pH = 7.5) for 1 h and then incubated overnight at 4 °C with primary antibodies diluted in TBS. Before the hybridization with primary antibodies, membranes were cut at a desirable range of protein mass (kDa) based on the Thermo Scientific Page Ruler Plus Prestained Protein Ladder (#26619). We used anti-CAT (Santa Cruz, CA, USA, sc-271803, 1:500) (Molecular Weight (MW) of 64 kDa, detected between 55 and 95 kDa), anti β-actin (Santa Cruze, sc-47778, 1:1000) (MW of 43 kDa, detected between 36 and 55 kDa), anti-SOCS3 (Thermo Fisher Scientific, Cat# PA1-29534, RRID:AB_2193284, 1:400) (MW of 30 kDa, detected between 36 and 28 kDa), anti-IL-6 (Santa Cruz, sc-57315, 1:500, Abcam ab6672, 1:500) (MW of 21 kD, detected between 36 and 17 kD), anti-CuZnSOD (Santa Cruz, sc-101523, 1:1000) (MW of 23 kD, detected between 28 and 17 kD). Furthermore, the membranes were washed in TBS with Tween (TBST) (pH = 7.5) and incubated in goat anti-mouse (Millipore, Cat# 12-349, RRID: AB_390192, 1:10000) or goat anti-rabbit (Sigma Aldrich, Cat# A9169, RRID: AB_258434, 1:30000) HRP-conjugated IgG antibodies for 1 h. Using Immobilon Western, Chemiluminescent HRP Substrate (Millipore, Burlington, MA, USA, WBKLS0100), antigen–antibody complexes were visualized and detected on the iBright CL 1500 instrument, accompanied by ImageJ software, version number: 2006.02.01.

### 4.8. Statistical Analyses

Statistica 12 was used for the statistical analysis of data. To ascertain whether the distribution of the data was normal, the Shapiro–Wilk test was applied. Levene’s test determined whether the groups’ variances were comparable. FST results were analyzed by three-way repeated measures ANOVA with stress and Olz set as independent factors (control vs. CSIS vs. Olz) and time (0-baseline, 3 weeks, and 6 weeks) set as a repeated measure, which was followed by Duncan’s post hoc test (*n* = 6 animals per group). Immunofluorescence and Western blot results were analyzed using a two-way ANOVA (the factors were drug treatment (levels: vehicle and Olz) and stress (controls and CSIS)), which was followed by Duncan’s post hoc test (*n* = 5–6 animals per group). Data are shown as mean ± standard error of mean (SEM). The statistical significance was set at *p* < 0.05.

## Figures and Tables

**Table 1:** Statistically significant results of immobility, swimming, and climbing time in the forced swim test (FST) in controls (Cont), chronic social isolation (CSIS), and olanzapine-treated CSIS (CSIS + Olz) rats at baseline (0 weeks) and at the end of the 3rd and 6th weeks. Significant differences between groups obtained by a three-way repeated measures ANOVA followed by Duncan’s post hoc test are indicated as follows: immobility time—CSIS (3 weeks) vs. CSIS (baseline), ** *p* < 0.01; CSIS (6 weeks) vs. CSIS (baseline), *** *p* < 0.001; CSIS + Olz (6 weeks) vs. CSIS (6 weeks), ^##^ *p* ≤ 0.01; swimming time—CSIS (3 weeks) vs. CSIS (baseline) * *p* < 0.05; CSIS (6 weeks) vs. CSIS (baseline), * *p* < 0.05; CSIS + Olz (6 weeks) vs. CSIS (6 weeks), ^##^ *p* < 0.01; climbing time—Controls (6 weeks) vs. Controls (baseline), ** *p* < 0.01; CSIS (3 weeks) vs. CSIS (baseline), ** *p* < 0.01; CSIS (6 weeks) vs. CSIS (baseline), *** *p* < 0.001. The results are presented as mean ± standard error of mean (SEM) of 6 rats per each group.

Forced Swim Test									
	Immobility Time (Seconds)			Swimming Time (Seconds)			Climbing Time (Seconds)		
Group	0 week	3rd weeks	6th weeks	0 week	3rd weeks	6th weeks	0 week	3rd weeks	6th weeks
Cont							78 ± 13		43 ± 11 **
CSIS	57 ± 21	137 ± 37 **	169 ± 26 ***	182 ± 7	124 ± 27 *	109 ± 17 *	77 ± 17	39 ± 17 **	22 ± 11 ***
CSIS + Olz			85 ± 17 ^##^			186 ± 9 ^##^			

## Data Availability

All data were included in this manuscript. Additionally, the fluorescence images (a.o. JPG format) generated and/or analyzed during the current study are available on request from the corresponding author. However, the uncropped fluorescence images presented in the manuscript Figure 1, Figure 2, Figure 3 and Figure 4 are generated in the Supporting information files alongside the manuscript. The original Western blots used in this study are also available in the Supplementary Materials files.

## Supplementary Materials

The supporting information can be downloaded at https://www.mdpi.com/article/10.3390/ijms242417181/s1.
