# Supplementary material for: Olanzapine Effects on Parvalbumin/GAD67 Cell Numbers in Layers/Subregions of Dorsal Hippocampus of Chronically Socially Isolated Rats
Source: Int J Mol Sci. 2023 Dec 6;24(24):17181. doi: 10.3390/ijms242417181 (PMC10743576; doi:10.3390/ijms242417181)

Supporting information files

Figure S1A is generated from the following materials:

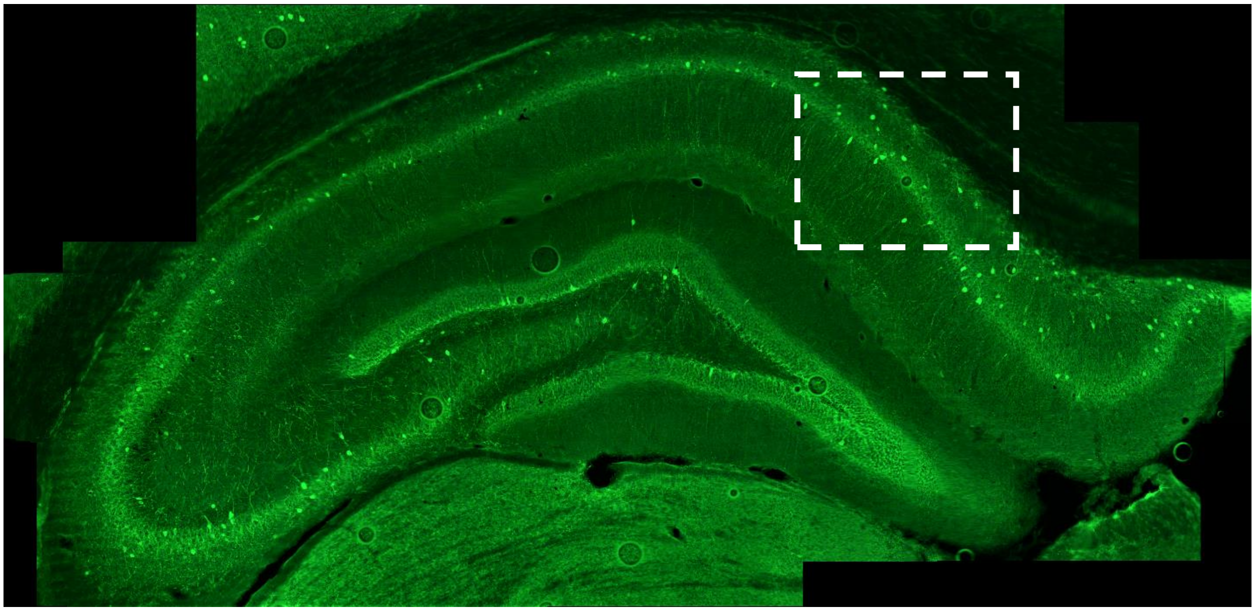

dHIPP-Cont

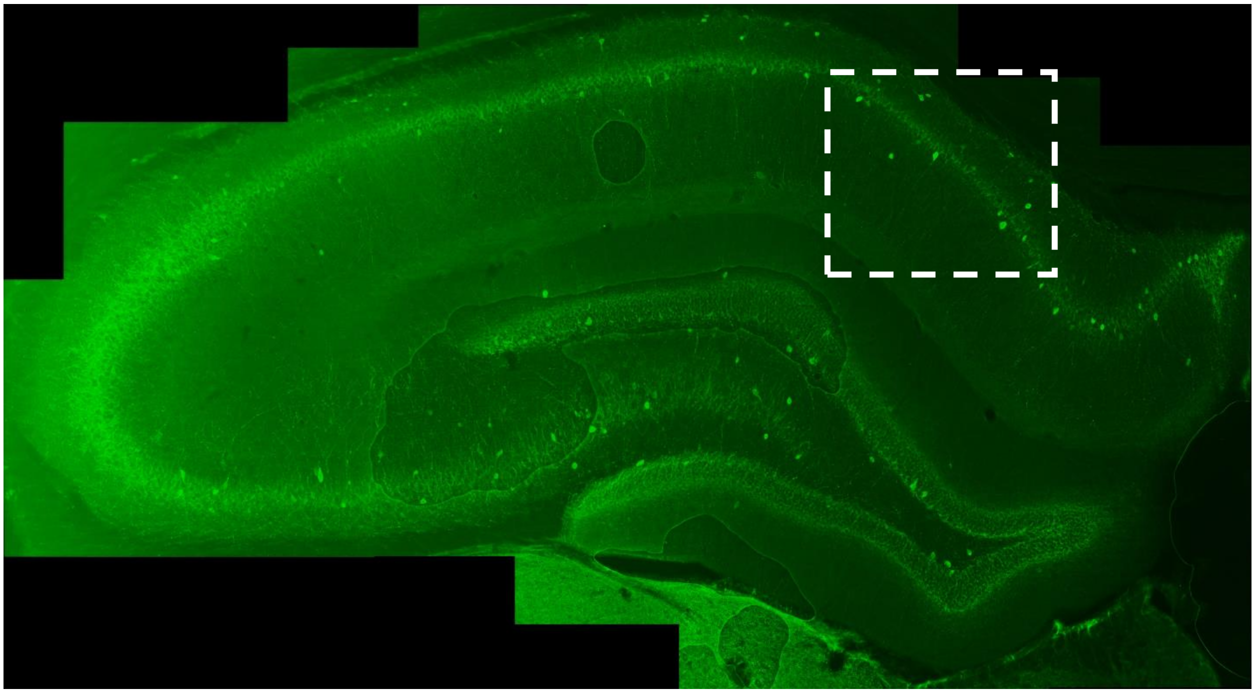

dHIPP-Cont + Olz

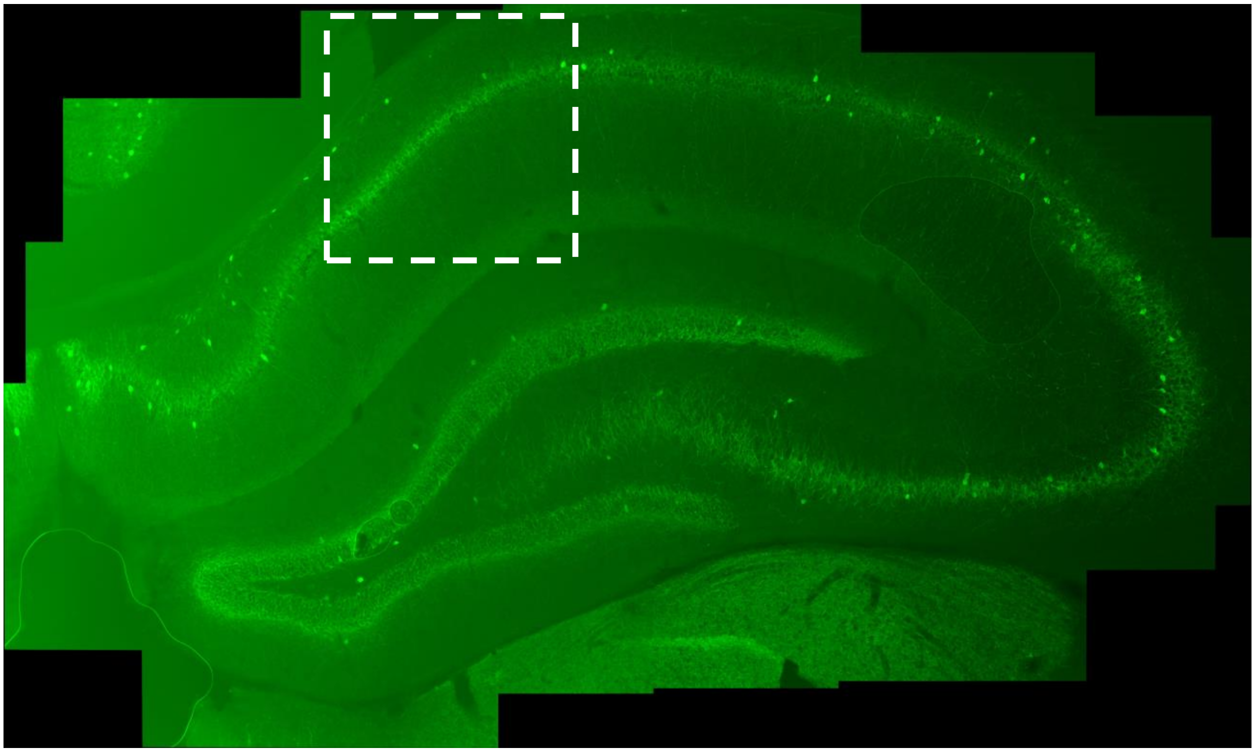

dHIPP-CSIS

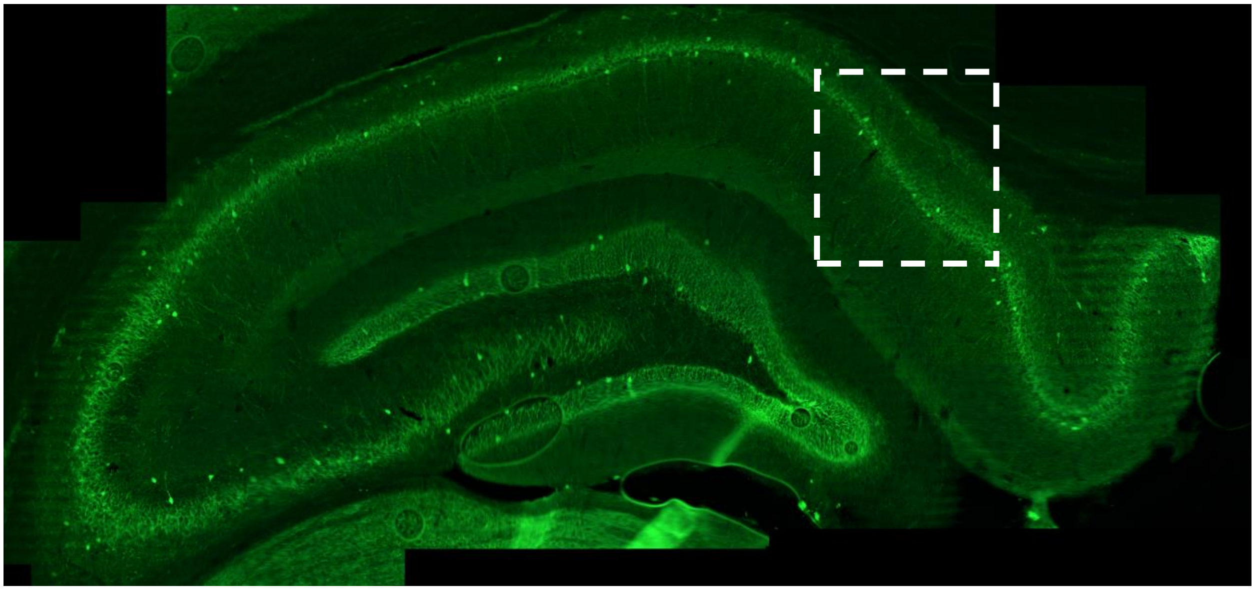

dHIPP- CSIS + Olz

Figure S1 B is generated from the following materials:

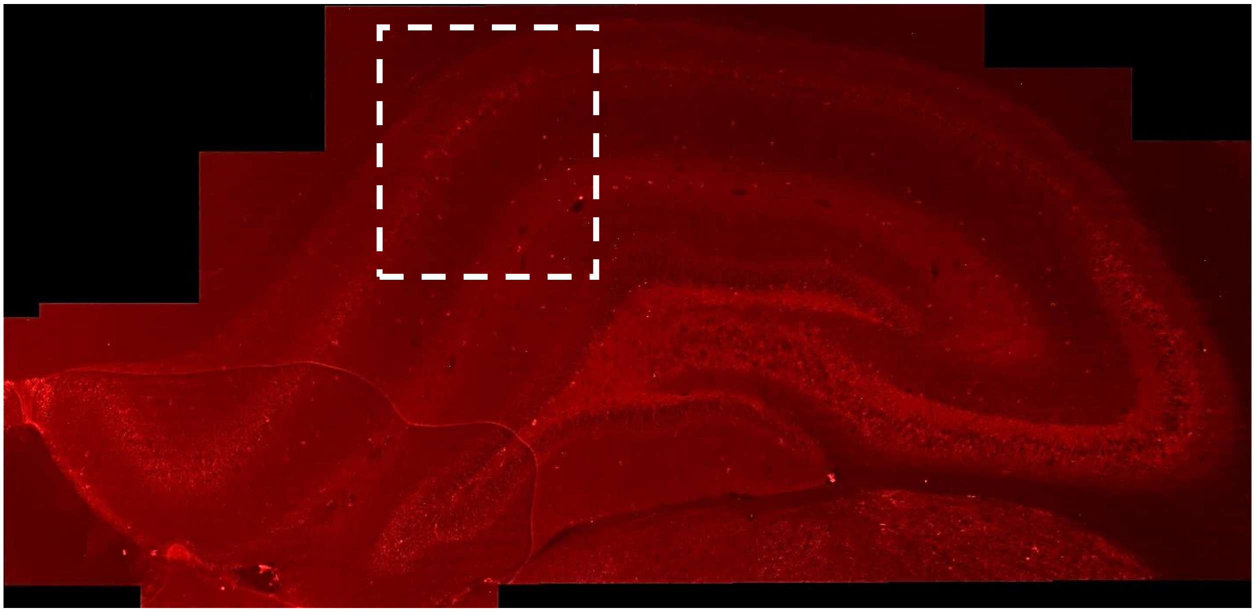

dHIPP – Cont

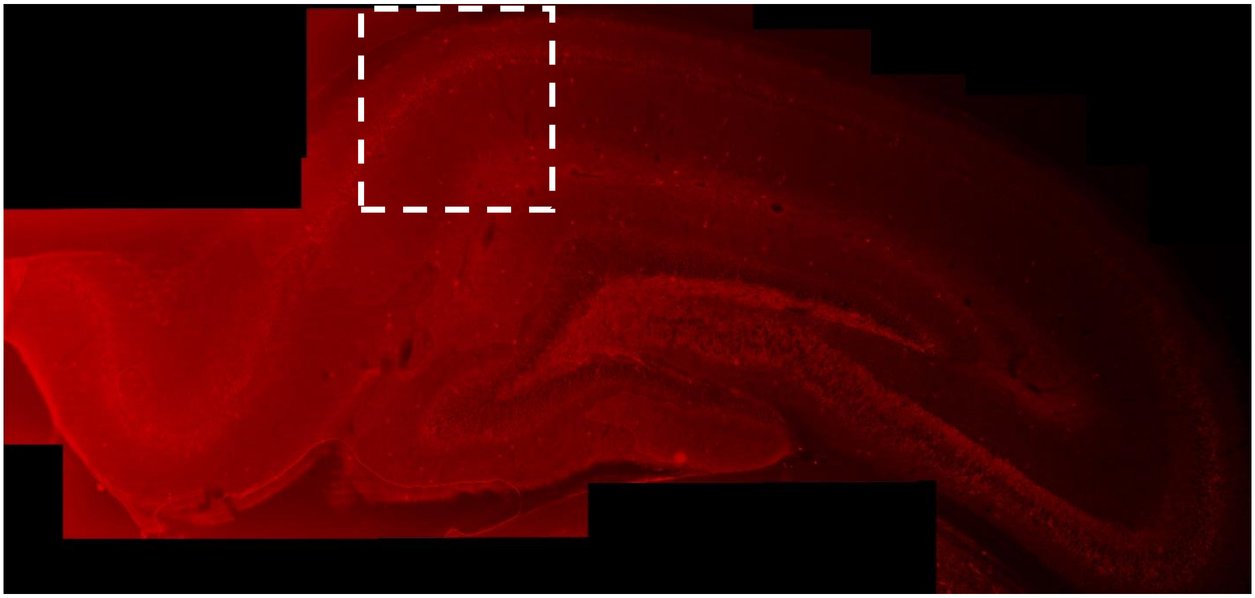

dHIPP- Cont + Olz

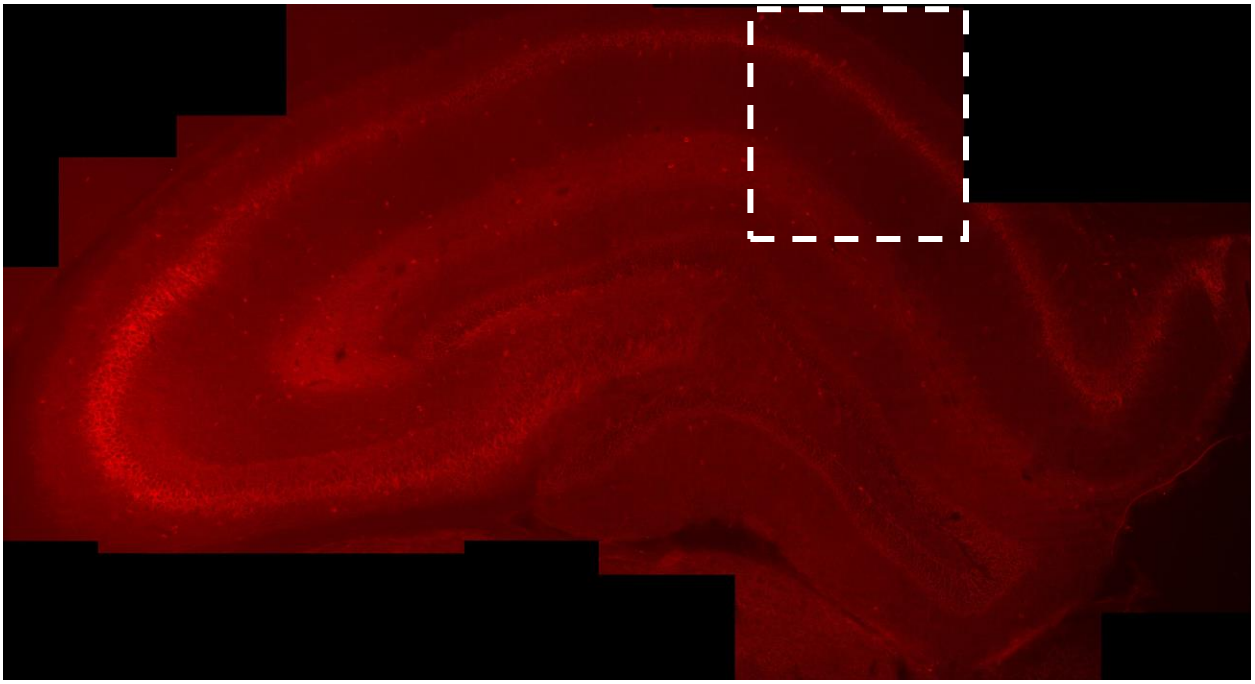

dHIPP- CSIS

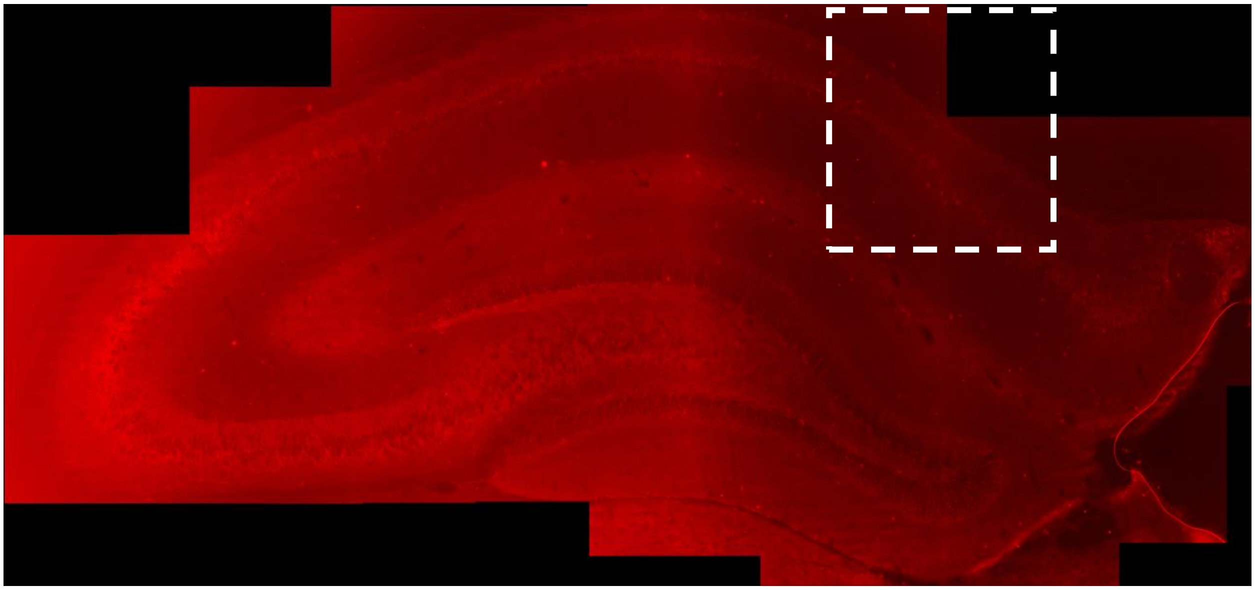

dHIPP- CSIS + Olz

Figure S2A is generated from the following materials:

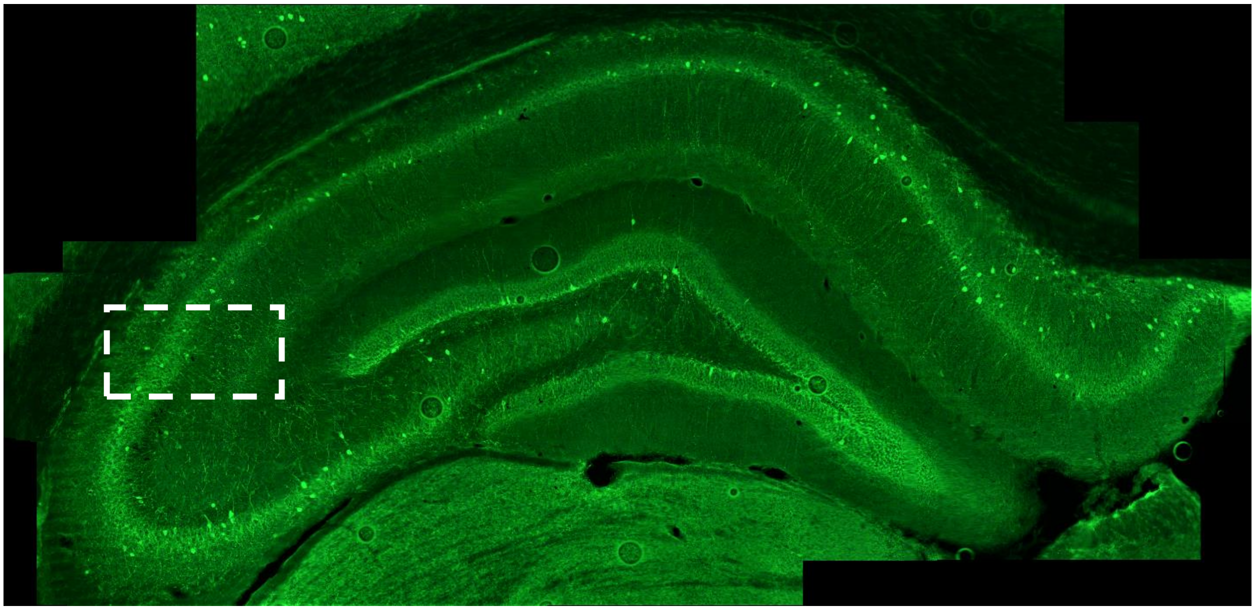

dHIPP- Cont

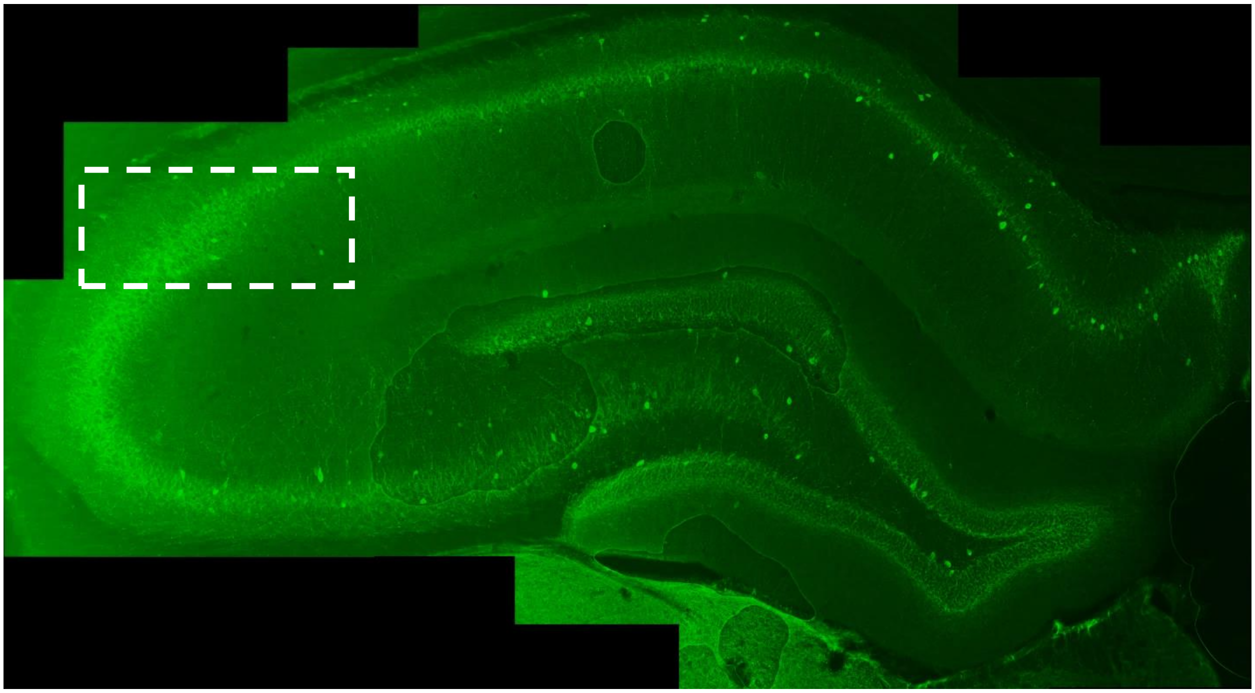

dHIPP- Cont + Olz

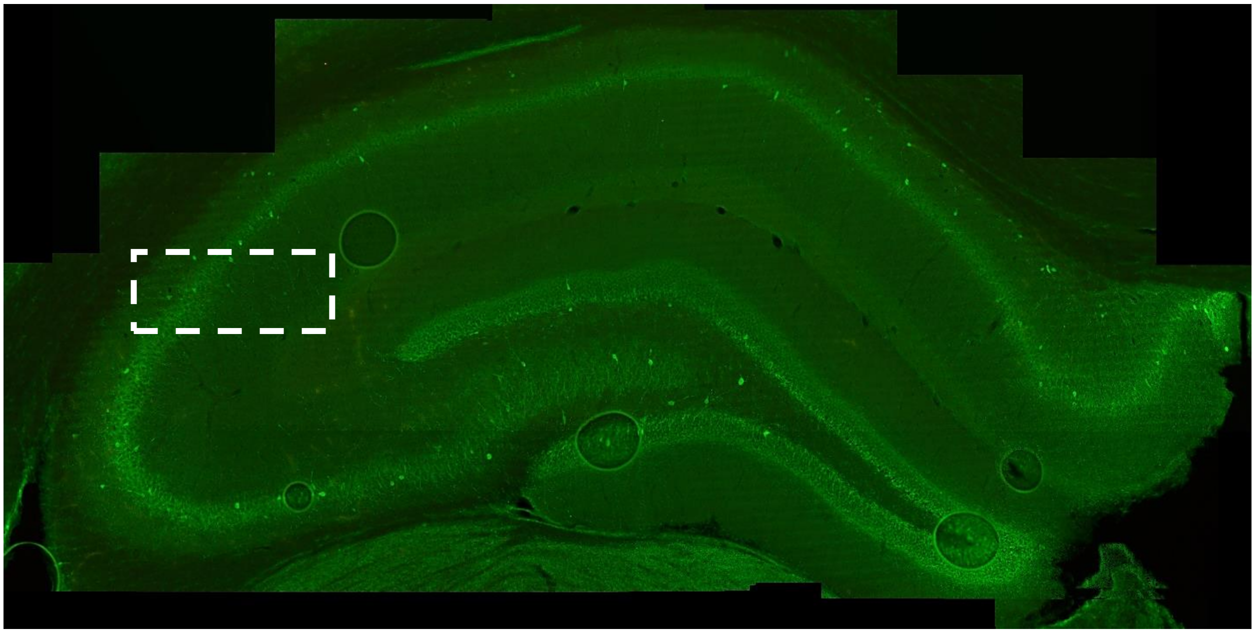

dHIPP- CSIS

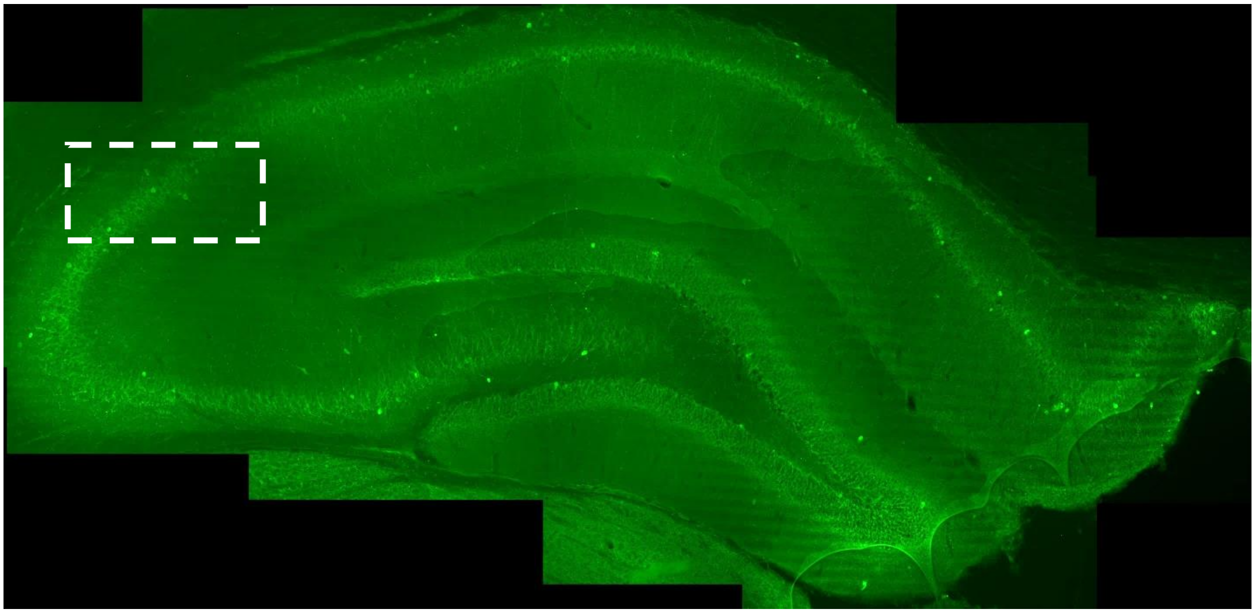

dHIPP- CSIS + Olz

Figure S2 B is generated from the following materials:

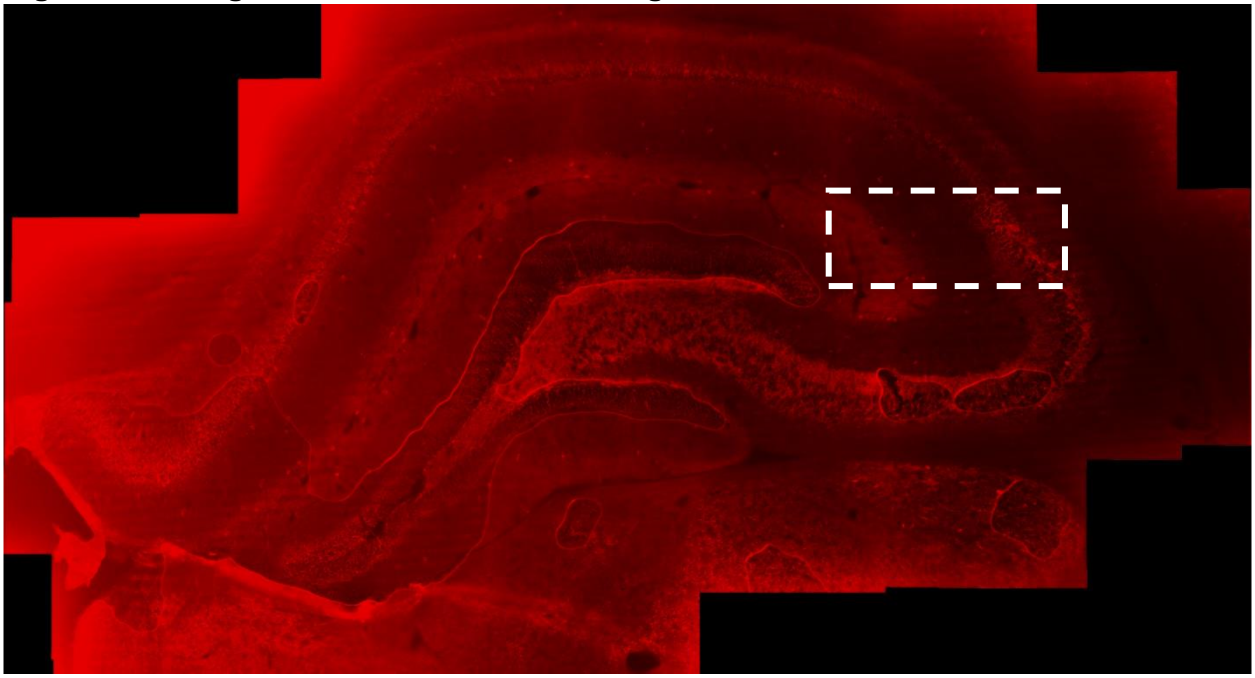

dHIPP- Cont

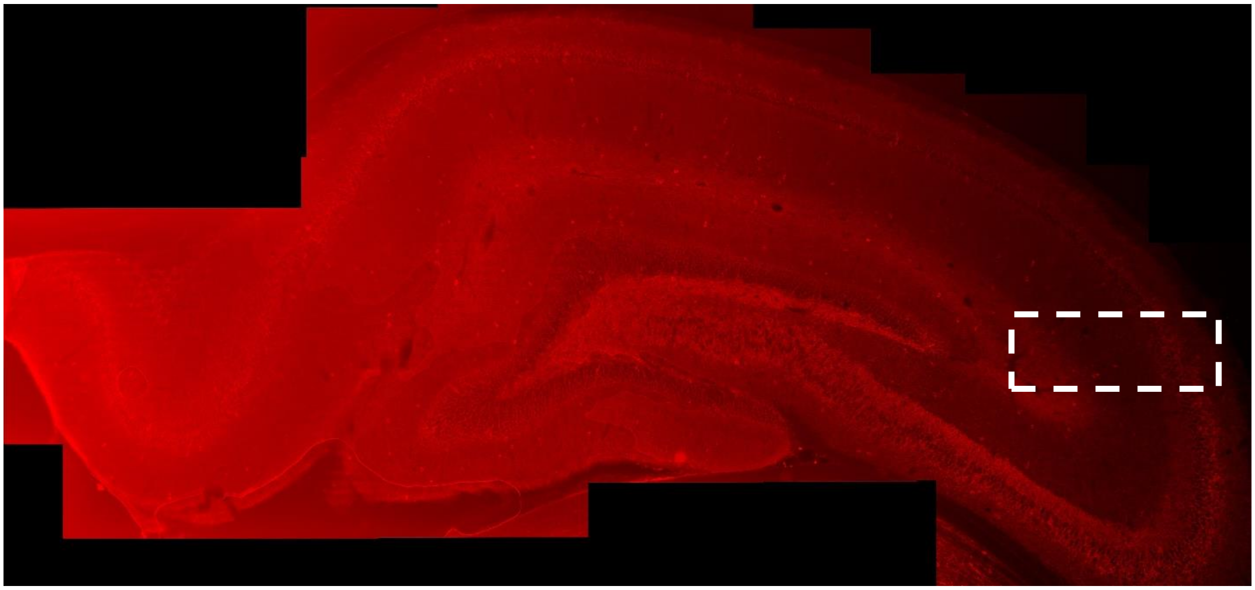

dHIPP- Cont + Olz

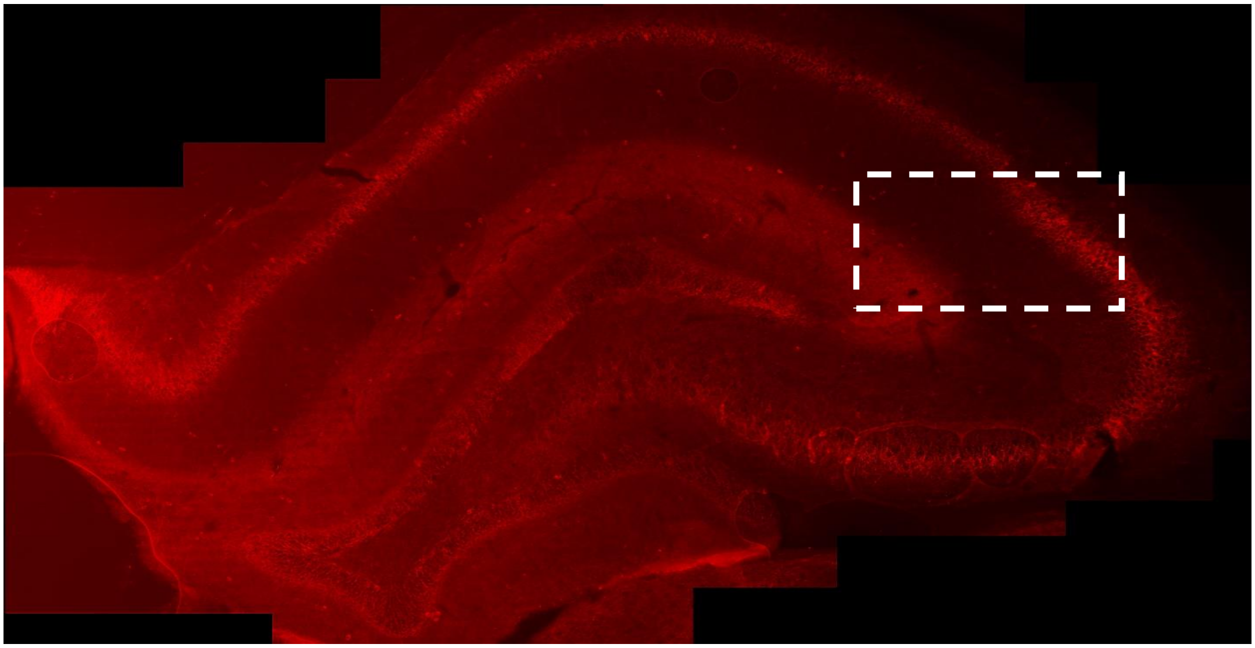

dHIPP- CSIS

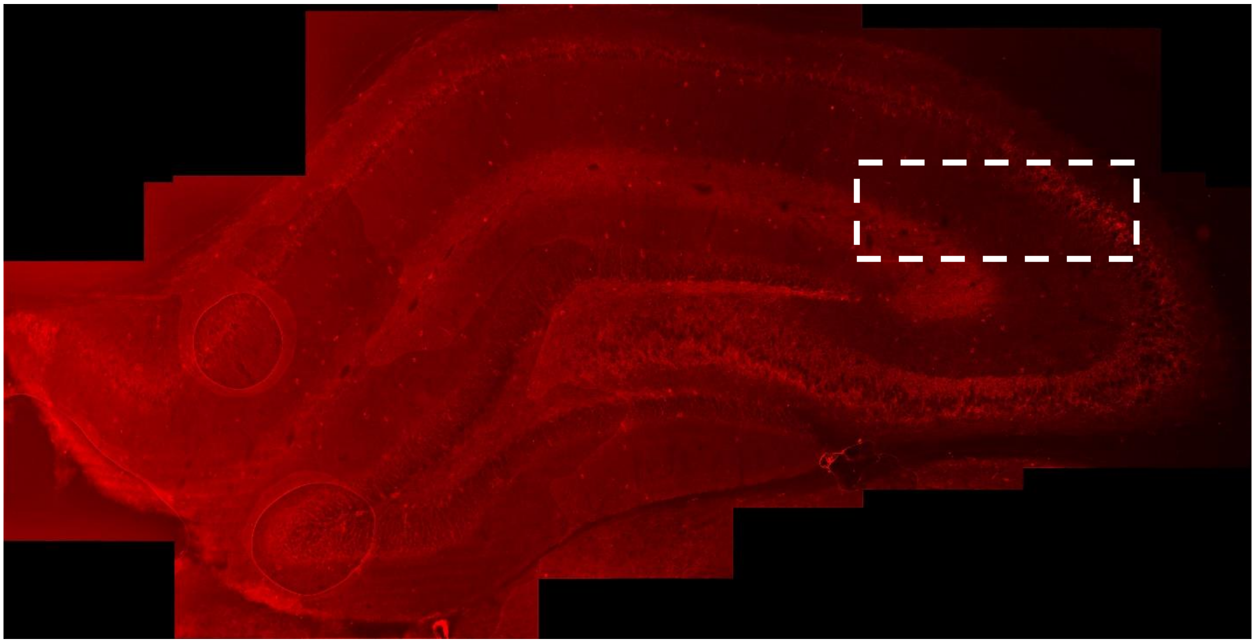

dHIPP – CSIS + Olz

Figure S3 A is generated from the following materials:

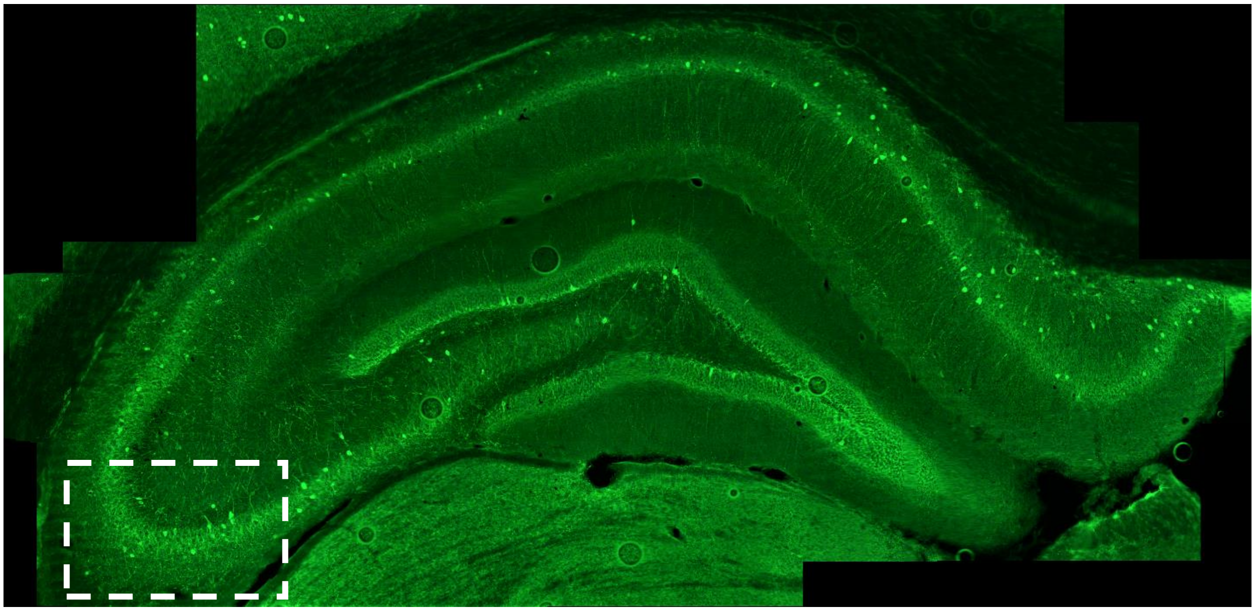

dHIPP- Cont

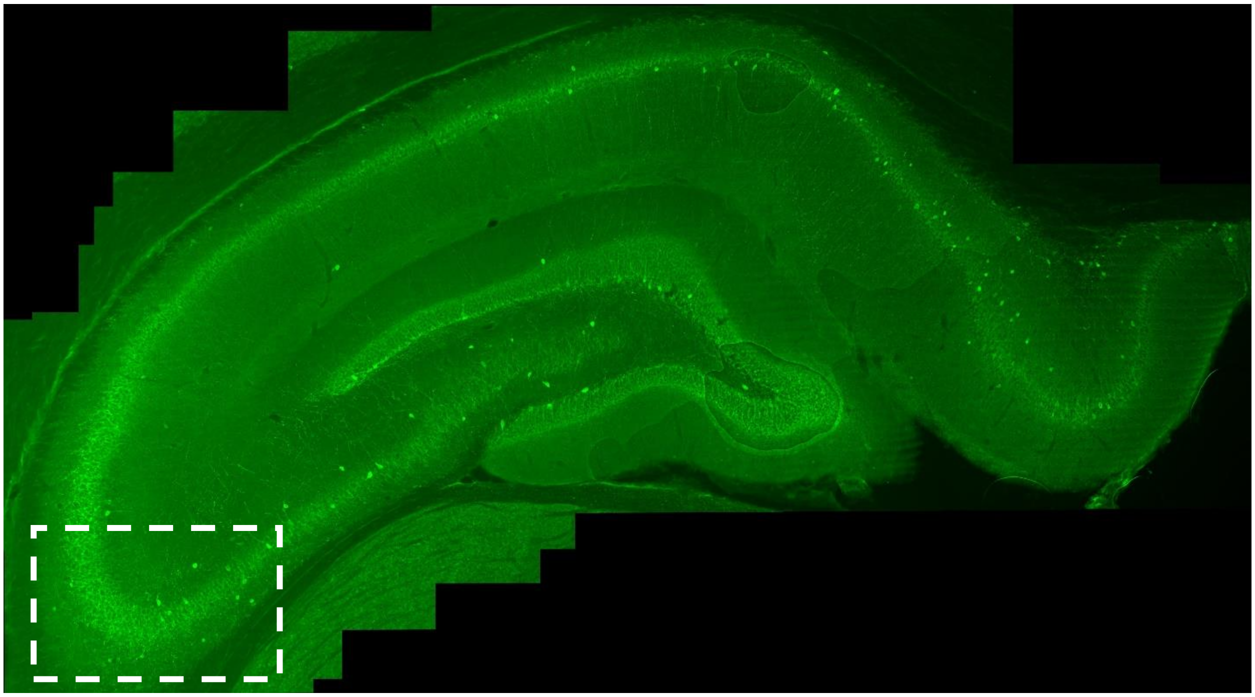

dHIPP- Cont + Olz

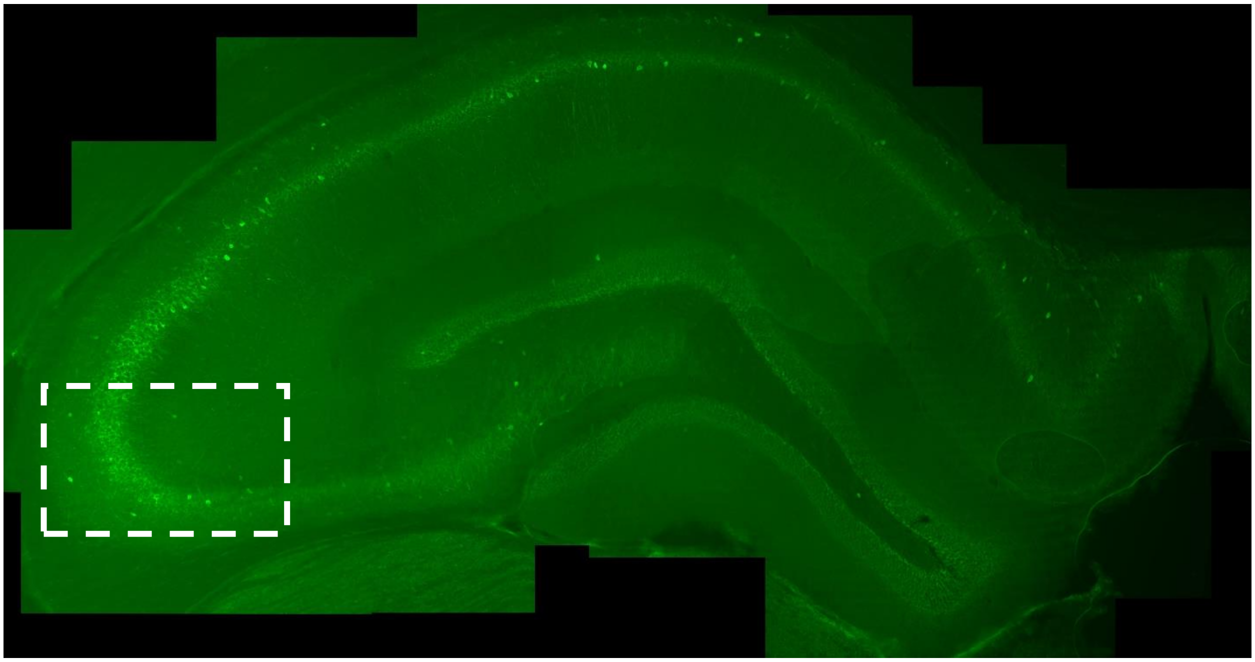

dHIPP- CSIS

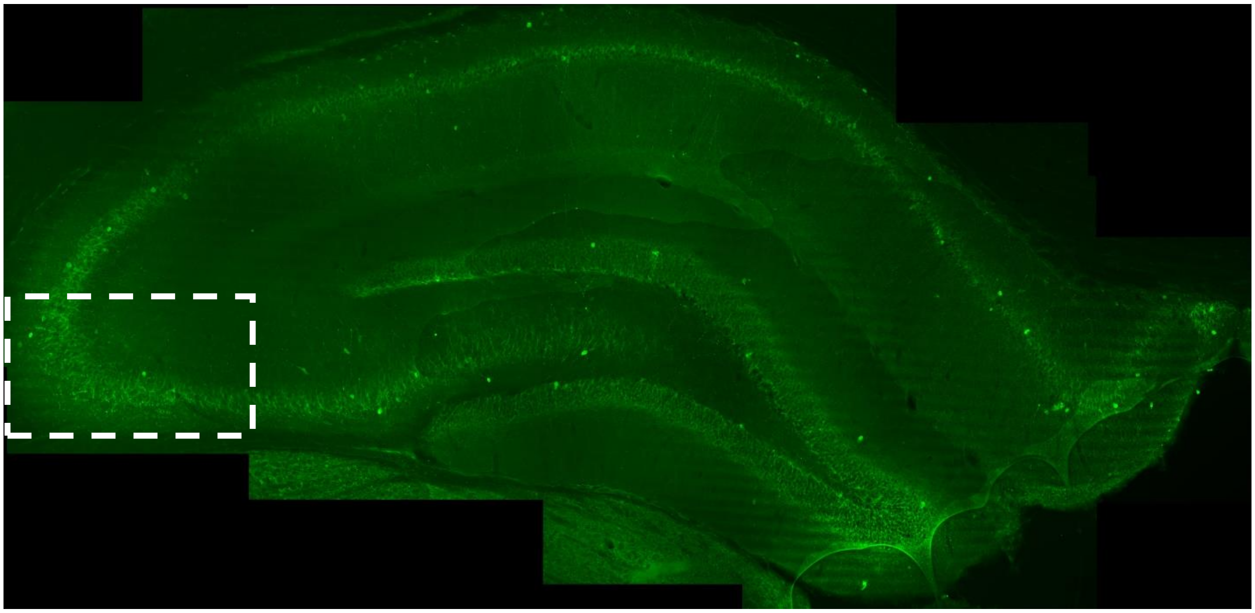

dHIPP- CSIS

Figure S3 B is generated from the following materials:

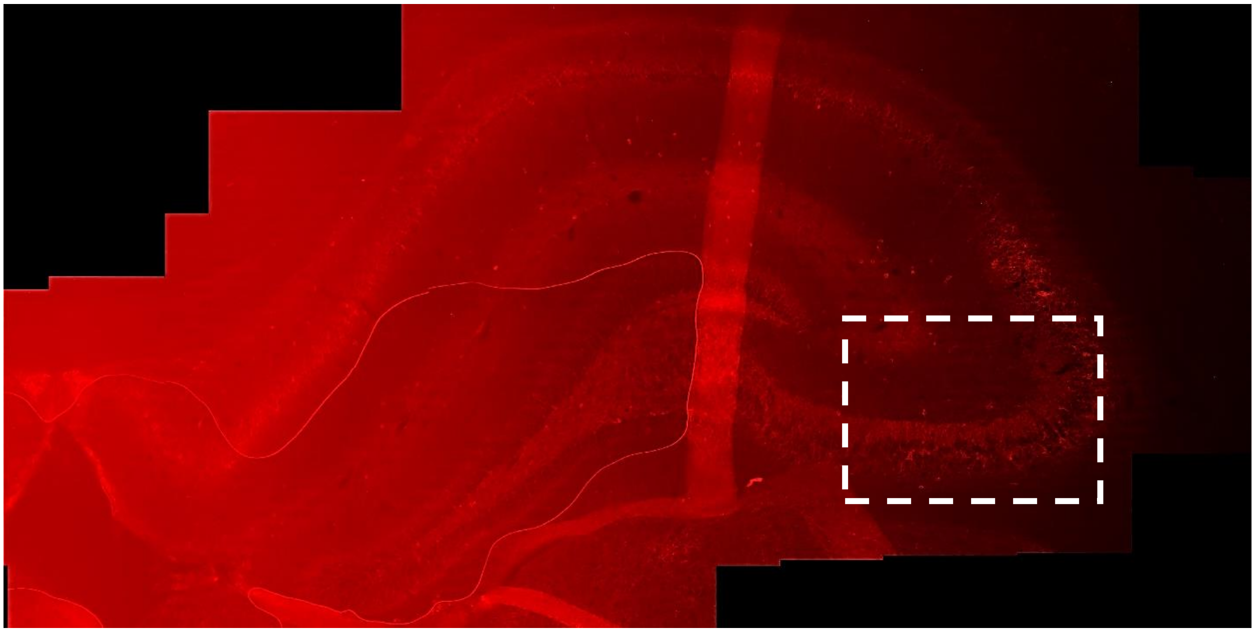

dHIPP- Cont

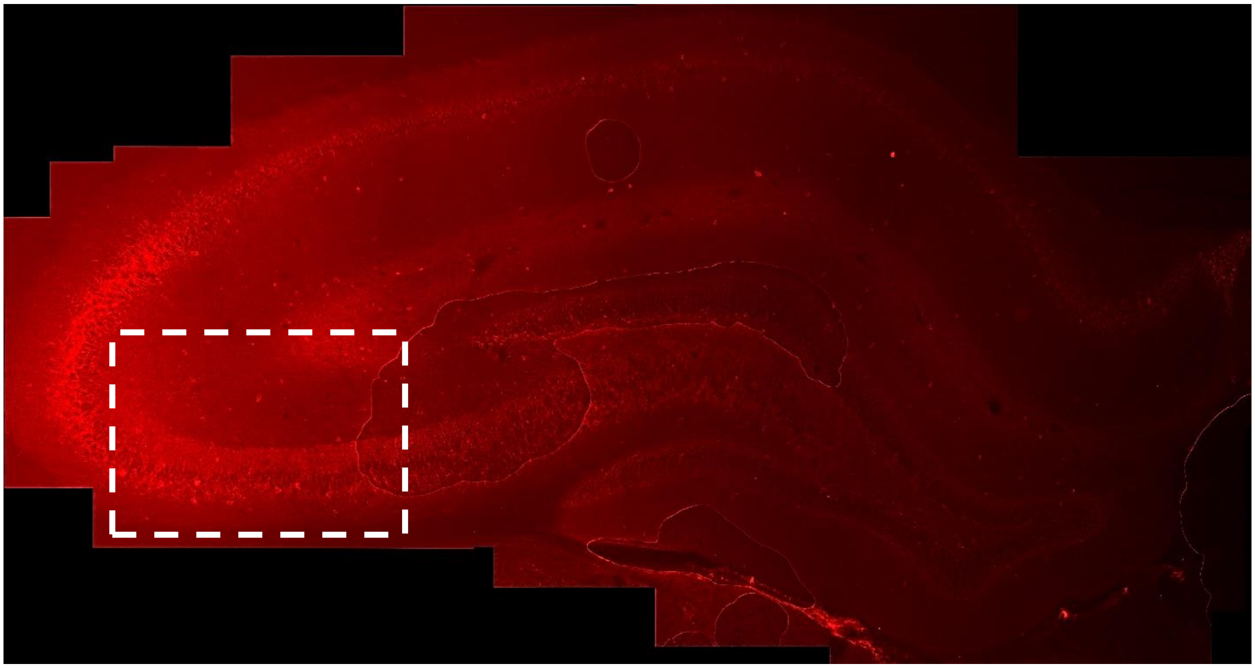

dHIPP- Cont + Olz

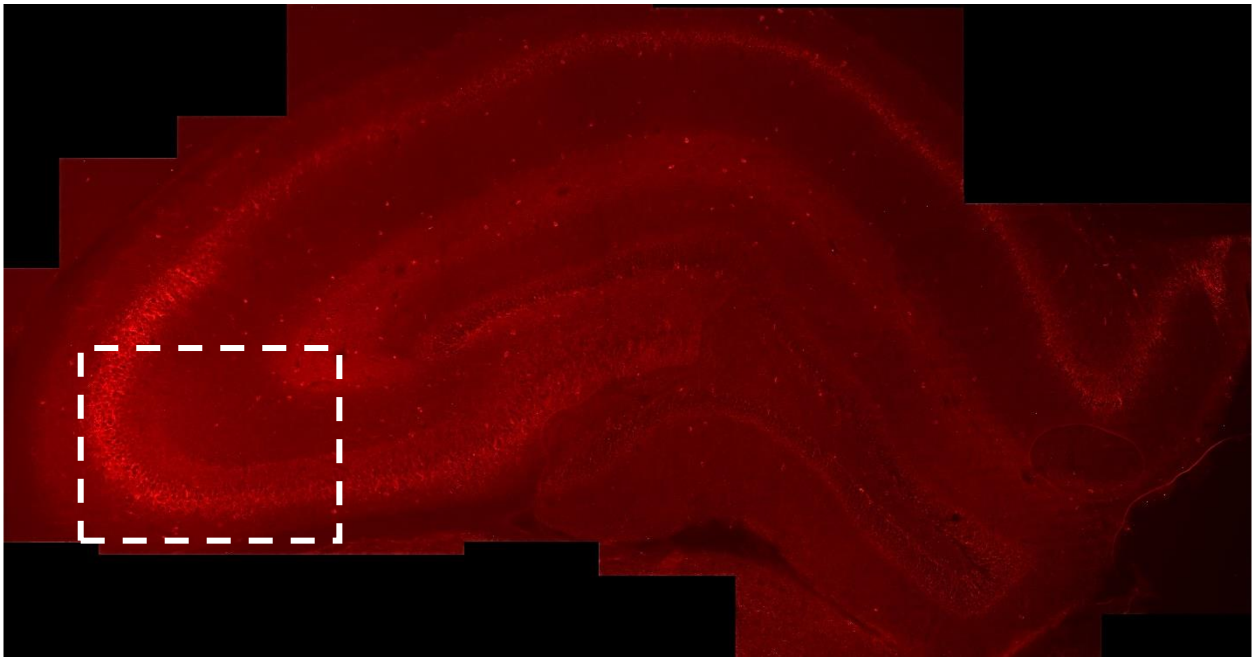

dHIPP – CSIS

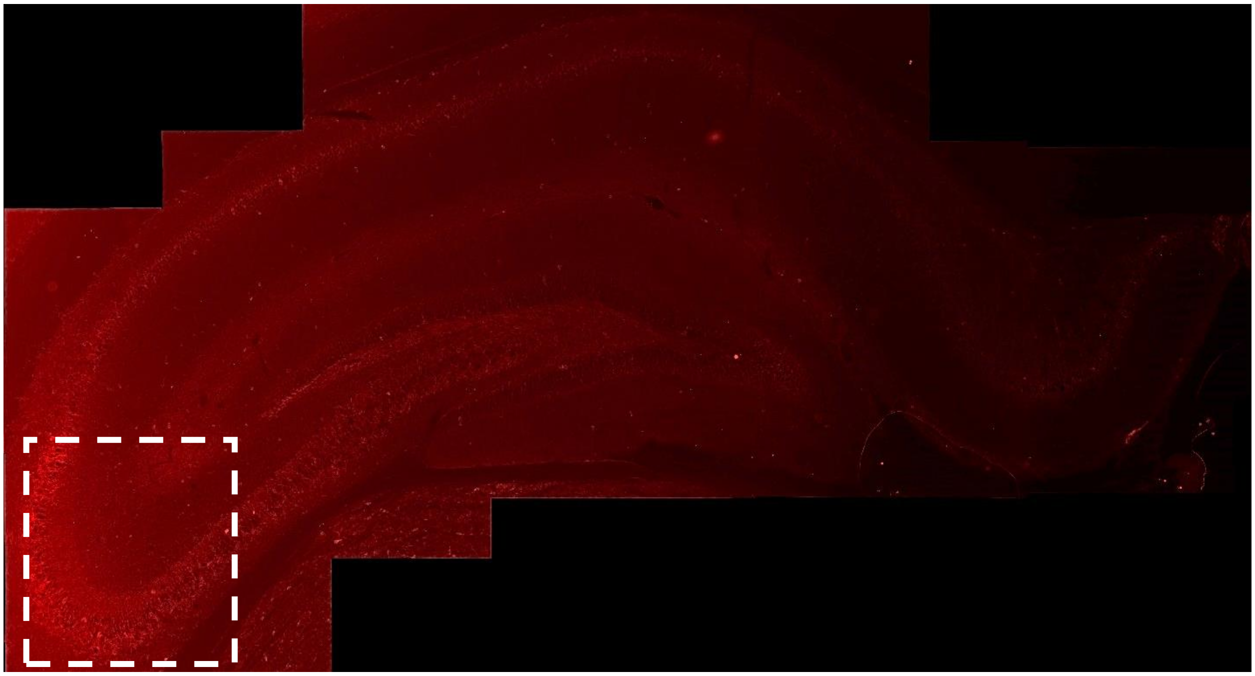

dHIPP- CSIS + Olz

Figure S4 A is generated from the following materials:

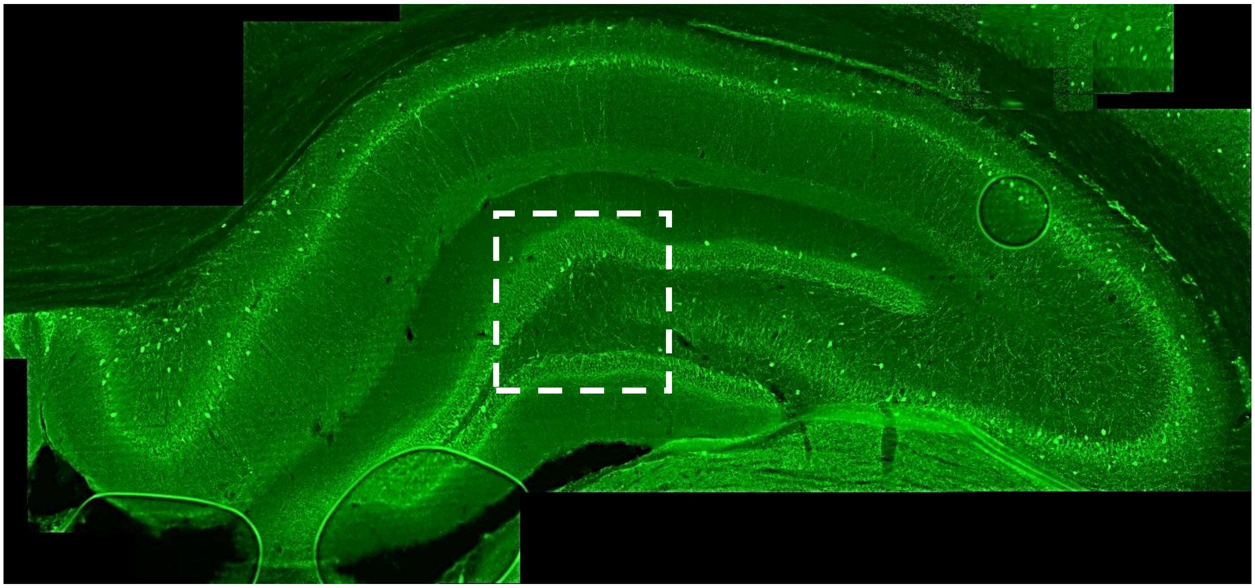

dHIPP – Cont

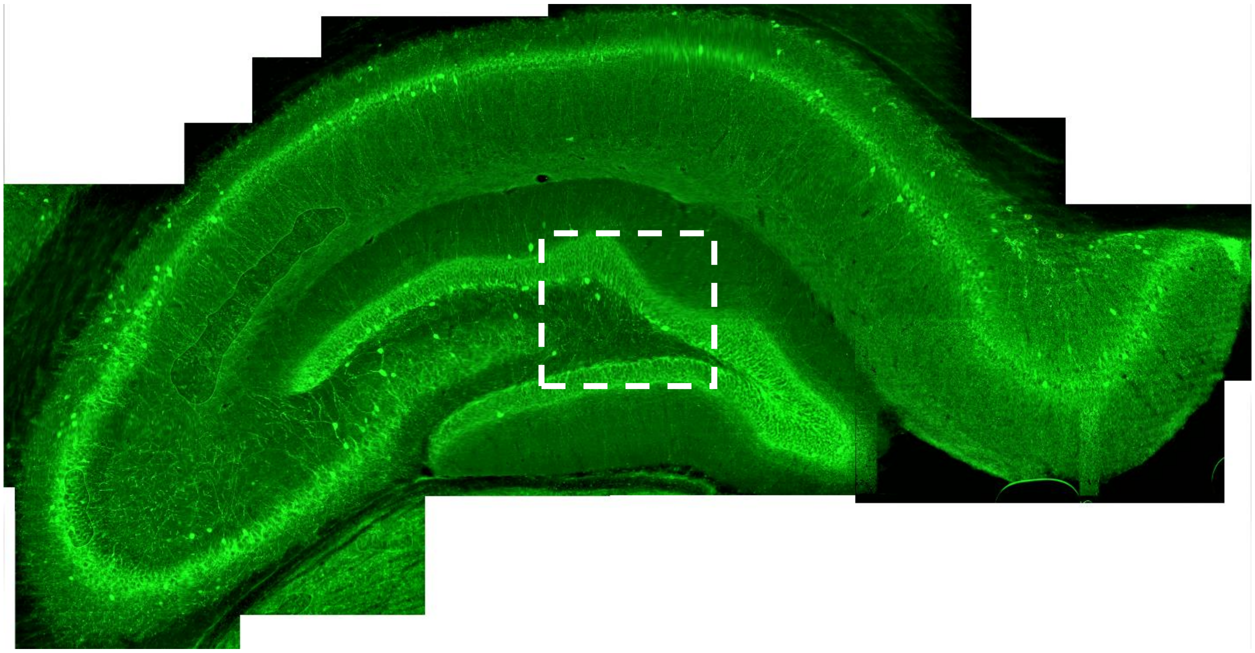

dHIPP- Cont + Olz

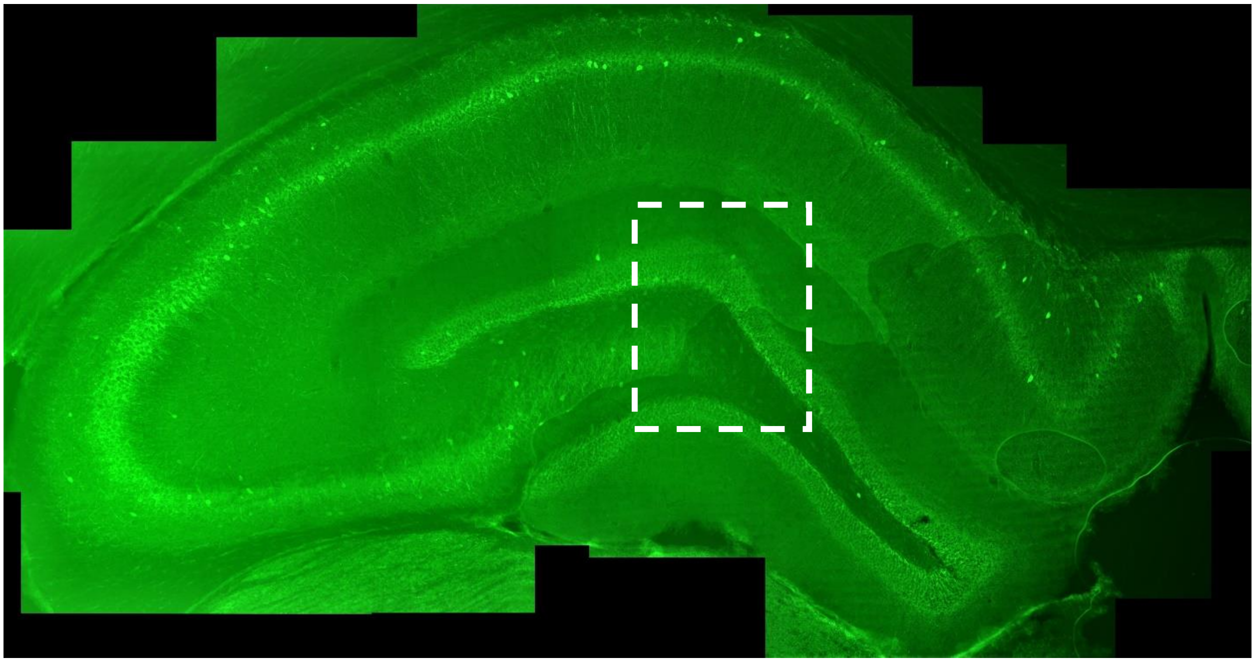

dHIPP- CSIS

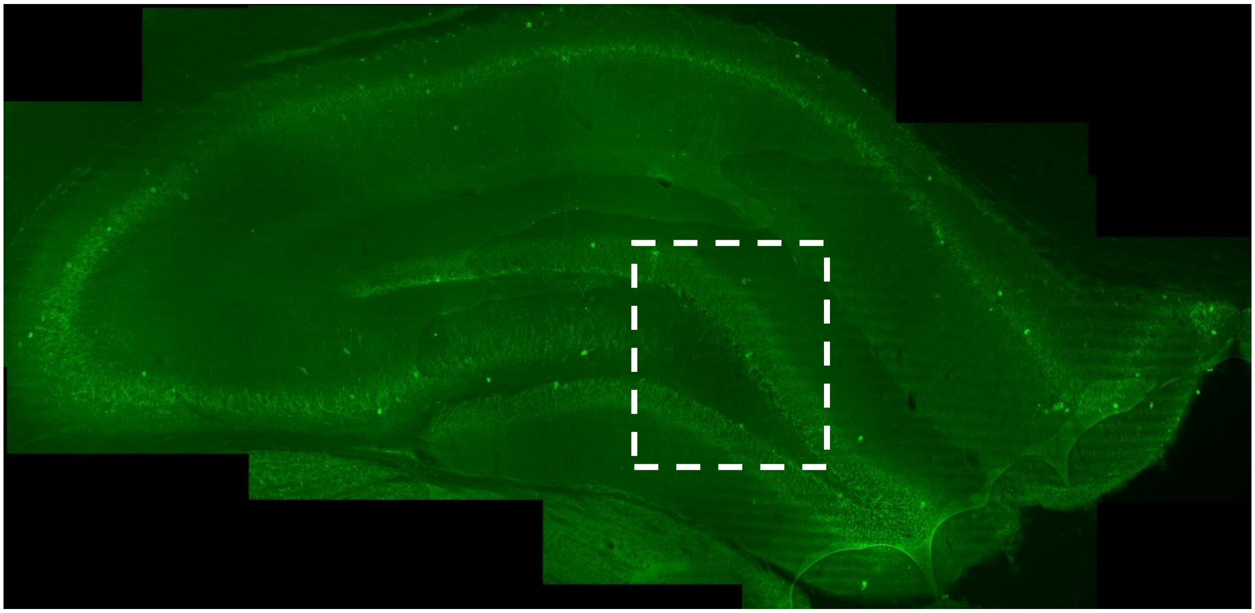

dHIPP- CSIS + Olz

Figure S4 B is generated from the following materials:

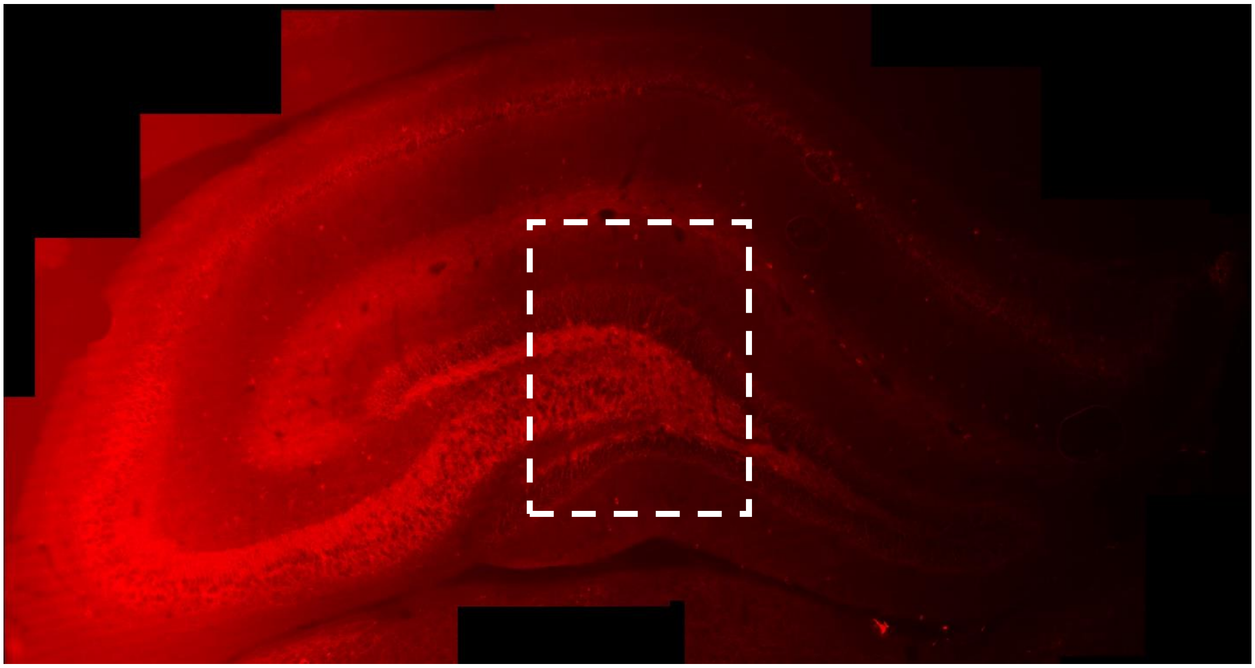

dHIPP- Cont

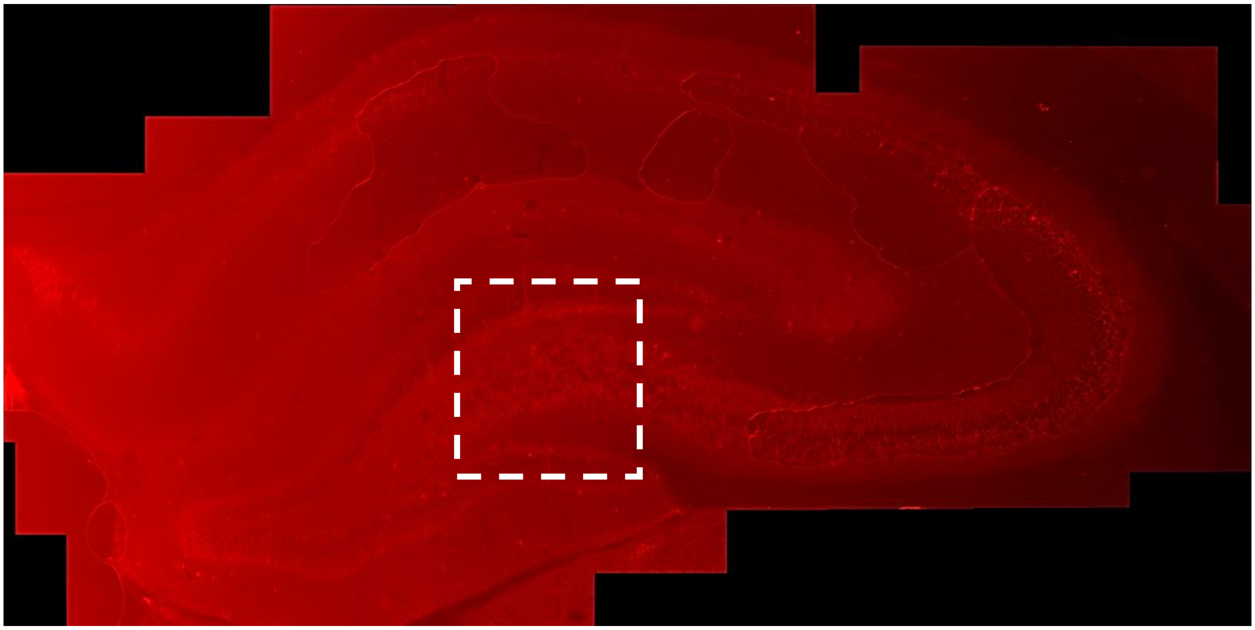

dHIPP – Cont + Olz

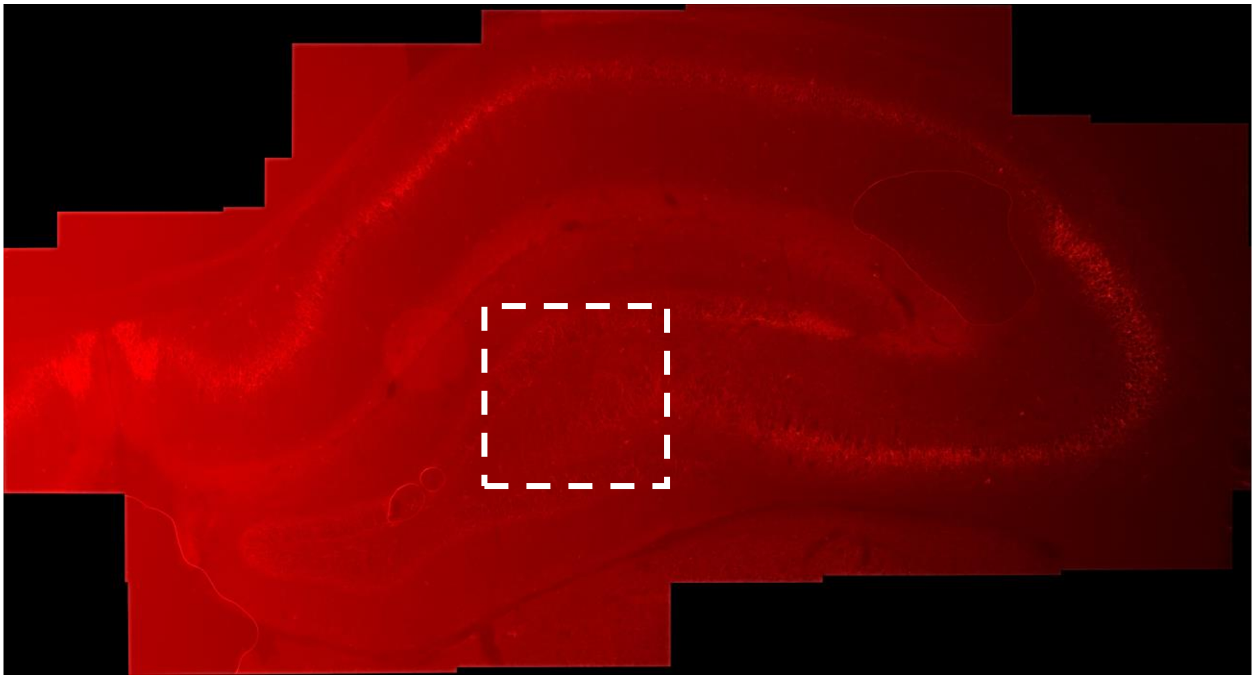

dHIPP- CSIS

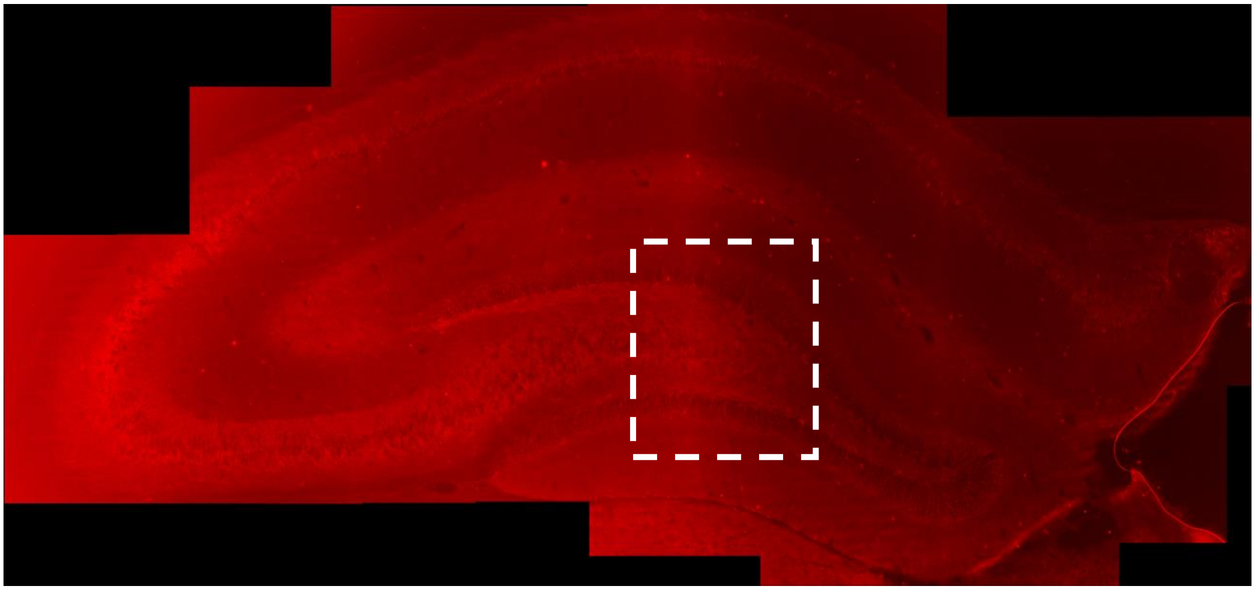

dHIPP- CSIS + Olz

The original western blotting of Figure 6 can be found below

1-Cont

2-Cont + Olz

3-CSIS

4-CSIS + Olz

The mebranes with dashed-border rectangle are represantive cropped blots from Figure 6 A-D. In the western blotting experiment we detected several proteins with different molecular weights on one entire membrane. This is the reason why each protein is on separate peace of the membrane.

The protein expression of CuZnSOD in the cytosol of HIPP

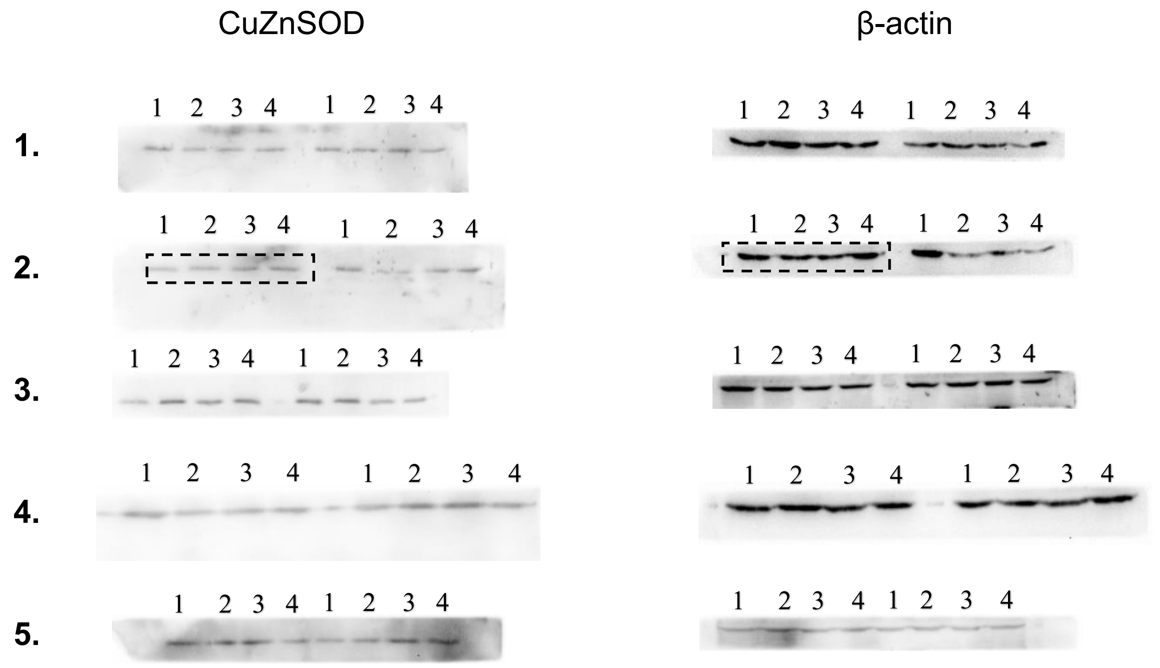

The protein expression of Catalase (CAT) in the cytosol of HIPP

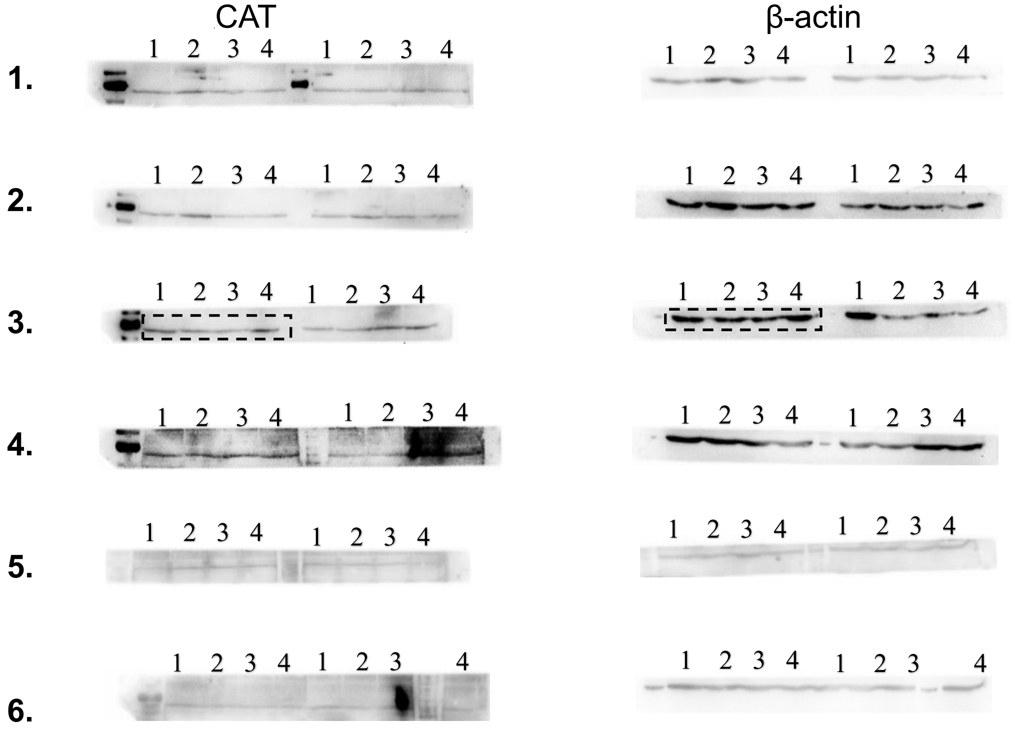

The protein expression of IL-6 in the cytosol of HIPP

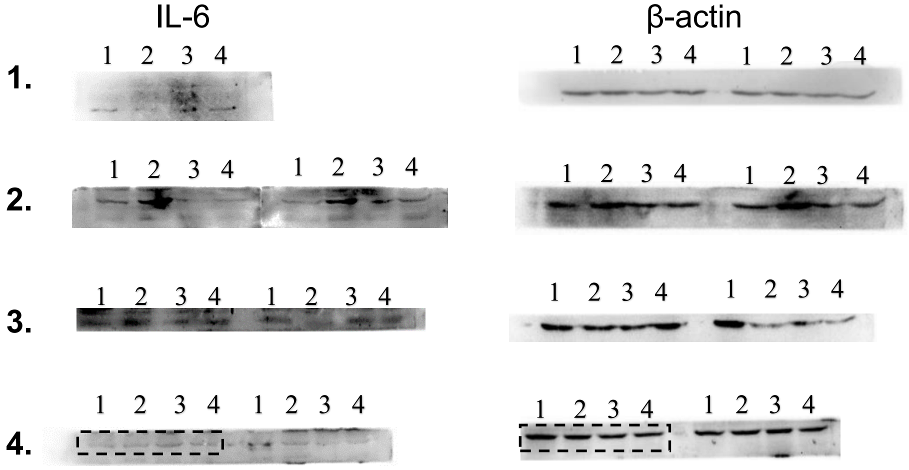

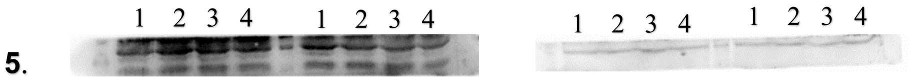

The protein expression of SOCS3 in the cytosol of HIPP

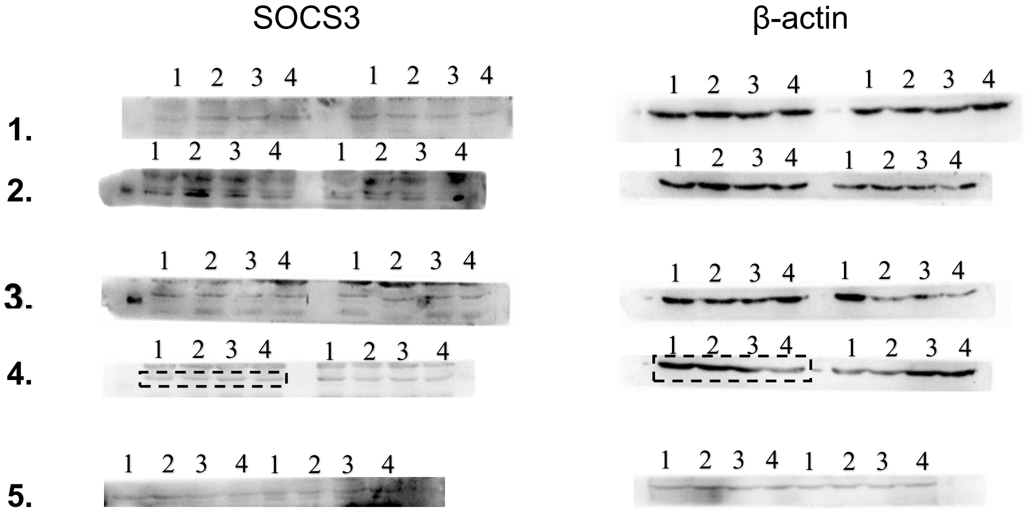

Supplement: Supplementary file 1 [file ijms-24-17181-s001.zip › ijms-2664819-supplementary.pdf]
